# Analysis of Antioxidant Phytochemicals and Anti-Inflammatory Effect from *Vitex rotundifolia* L.f

**DOI:** 10.3390/antiox11030454

**Published:** 2022-02-24

**Authors:** DucDat Le, Sanghee Han, Jeongjun Ahn, Jayeon Yu, Chang-Kwon Kim, Mina Lee

**Affiliations:** College of Pharmacy and Research Institute of Life and Pharmaceutical Sciences, Sunchon National University, 255 Jungangno, Suncheon 57922, Jeonnam, Korea; ddle@scnu.ac.kr (D.L.); 1210092@s.scnu.ac.kr (S.H.); rnlghs5738@s.scnu.ac.kr (J.A.); 1210093@s.scnu.ac.kr (J.Y.); ckkim2149@scnu.ac.kr (C.-K.K.)

**Keywords:** *Vitex rotundifolia*, DPPH-HPLC, analytical method, antioxidant, NO production

## Abstract

An extraction method using 80% EtOH was selected and applied to obtain the total extracts from leaves, flowers, fruits, twigs, and roots of *Vitex rotundifolia* L.f. based on the antioxidant activity-guided experiments. Subsequently, total extract from each part of *V*. *rotundifolia* was successfully partitioned into fractions, which were evaluated for their antioxidant and anti-inflammatory properties via DPPH, ABTS, and NO assays, respectively. Among them, EtOAc (E) and *n*-butanol (B) fractions showed the potent antioxidant activity and the methylene chloride (MC) fractions of roots, leaves, and fruits that exhibited strong scavenging activity on DPPH and ABTS radicals. In the anti-inflammatory assay, *n*-hexane (H) and MC fractions of leaves potently inhibited NO production in LPS-stimulated RAW264.7 cells, followed by E fractions derived from fruits, flowers, twigs, and roots, along with B fractions from flowers and twigs. Additionally, a comprehensive HPLC-decoupled MS profiling was established and validated using seven isolated marker compounds (**1**–**7**), which were identified by analysis of their UV, NMR, and MS data. The established method was also applied for quantification of these marker compounds in each organ collected from different locations, and to assess their antioxidant capacity by a screening DPPH-HPLC method. Principal component analysis suggested the botanical organs from this plant correlated with the marker compound contents in association with bioactivity. The study results are a prelude to further studies involving the active fractions and provide a comprehensive insight into the functional products of this plant against oxidative diseases.

## 1. Introduction

Oxidative stress associated with free radicals and reactive metabolites may be attributed to increased levels of free radicals or decreased antioxidant concentration. Free radicals contain unpaired electrons, which are unstable and reactive in oxidative reactions involving other reactive species such as carbonyl (methylgyoxal, glyoxal) [1], nitrogen compounds (peroxynitrite, nitric oxide) [2], and sulfur [3], which may undergo a series of sequential reactions to generate abundant reactive metabolites. This process is associated with electron transfer reactions influencing the redox state of cells and the organism. The changed redox state further stimulates or inhibits activities of various signal proteins, resulting in altered signal transduction pathways to influence the fate of cells [4]. Oxidation may damage the biomolecules (DNA, lipids, and protein) in various diseases and conditions such as inflammation, aging, cancer, diabetes, Parkinson’s disease, and atherosclerosis [5,6,7].

It is necessary to identify the antioxidants that may prevent free radical formation by inhibiting enzymes catalyzing free radical formation via reduction of superoxide (allopurinol) or eliminating the catalytic activity associated with free radical formation. Further, antioxidants may also eliminate the reactivity of free radicals by transforming them into nonradical and nontoxic metabolites.

*Vitex rotundifolia* belongs to family Verbenaceae, and is found in tropical and temperate areas of Korea, Japan, Southeast Asia, and the Pacific islands [8]. It is also well-known as a medicinal plant of coastal Korea and China, was traditionally used for the treatment of colds, headaches, migraines, eye-pain, neuralgia, and premenstrual syndrome [9]. Furthermore, this plant is also use for the treatment of asthma, chronic bronchitis, and gastrointestinal infections, including bacterial dysentery and gastroenteritis diarrhea [10]. A previous study revealed that the total extracts from this plant exhibited the significant anti-inflammatory effects by down-regulated expression of inflammation-related genes [11,12]. Among them, casticin and aucubin, isolated from *V. rotundifolia* [13], exhibited the potential anti-inflammatory activity [14,15]. In addition, previous studies revealed that the total extract of twigs [16] and some isolated constituents, orientin and quinic acid compound, [17] derived from this plant exhibited potent antioxidant activity, which prompted us to perform appropriate studies. Despite the availability of commercial products derived from *V. rotundifolia*, no studies validating the contents using these marker compounds and antioxidant competence of the five organs of this plant are currently available.

In this study, biological assays including both enzyme-linked immunosorbent assay (ELISA) and direct analytical method using 1,1-diphenyl-β-picrylhydrazine-high performance liquid chromatography (DPPH-HPLC) experiment, and HPLC/mass spectrometry (MS) were used to analyze the antioxidant activity of active constituents [18] derived from five organs of this plant. The 80% EtOH and MeOH extracts of the five organs from this plant were screened to develop an extraction method based on DPPH and 2,2-azino-bis (3-ethylbenzothiazoline-6-sulfonic acid diammonium salt) (ABTS) assays. Subsequently, the total extracts of five different organs using 80% EtOH were obtained and successfully fractionated based on solvent-solvent system polarity. The antioxidant activity of fractions was further evaluated using ELISA. Furthermore, seven phytochemicals isolated from the leaves of this plant were used as the marker compounds to establish an analytical method. Then, the DPPH-HPLC experiment was conducted to screen the antioxidant components in the total extract using this established analytical method. The antioxidant capacities of these active components were further verified by an ELISA.

## 2. Materials and Methods

### 2.1. Plant Materials

The leaves, flowers, fruits, twigs, and roots of *V. rotundifolia* were collected in 2021 at Goheung (SCNUP 26_SBG-G1-5); Jeju (SCNUP 26_SBG-J1-4); Sinan (SCNUP 26_SBG-S1-4); and Busan (SCNUP 26_SBG-B1-4), Korea, and were identified by Prof. Mina Lee (College of Pharmacy, Sunchon National University). Their voucher specimens were deposited in the Laboratory of Pharmacognosy, College of Pharmacy, Sunchon National University (Suncheon, Korea).

### 2.2. Extraction Method

Firstly, five organs of *V. rotundifolia* collected at Goheung were shade dried at room temperature. Then, the dried material of each was coarsely powdered and was stored in a closed container for further studies. The powdered organs (1 g, each) were extracted with a volume of 40 mL of 80% EtOH and 100% MeOH by sonification for 90 min (min), respectively. The total extracts were kept in cool storage at 4 °C for further uses. These total extracts were used as materials to evaluate the antioxidant activity. Then, the extraction time was also investigated at 30, 60, 90, and 120 min, using the same volume of 80% EtOH to optimize the extraction time.

### 2.3. DPPH and ABTS Assays of Total Extracts and Fractions

The total extracts using 80% EtOH and 100% MeOH solvents extraction were tested for their radical scavenging activities on DPPH^•^ and ABTS^•+^ using our previous method [19]. Briefly, the antioxidant activity of each sample was evaluated by the ability of sample to scavenge the DPPH (Sigma-Aldrich, St Louis, MO, USA) radical. Each well contained 100 μL of sample volume which was diluted in ethanol to final concentrations (10 and 100 μg/mL) and 100 μL of DPPH (200 μM) in ethanol solution. Control was prepared with same conditions with the amount of sample replaced by addition of ethanol. All wells were mixed thoroughly and were incubated at room temperature for 30 min in shade. When DPPH reacts with an antioxidant sample, the color conversion of deep violet into light yellow was measured at 517 nm with a micro reader (Epoch, Biotek Instruments, Winooski, VT, USA). Additionally, 2,2′-azobis(2-aminopropane) dihydrochloride (7 mM) was mixed with ABTS (Sigma-Aldrich, Co.) at a concentration of 2.45 mM, and then reacted for 16 h at 4 °C. 50 µL quantity of the sample and 100 µL of the ABTS solution were mixed and reacted at room temperature for 20 min before being added to 96-well plates. The absorbance was measured at 734 nm. Ascorbic acid (Sigma-Aldrich, Co., St. Louis, MO, USA) (100 µg/mL) was used as the positive control for both assays.

The results were calculated as the percentage of DPPH and ABTS reduction between treated samples and control wells.

Based on the free radical scavenging activity examination, a solvent extract of 80% EtOH was employed for extraction, and these obtained total extracts were used for further study. Then, Goheung, Jeju, Sinan, and Busan samples of organs of *V. rotundifolia* were also prepared using a solvent extract of 80% EtOH and filtered through 0.45 μm filter for qualification of the marker compound contents by the same experimental conditions. Each of the five total extracts was suspended in water and successively partitioned with a series of organic solvents with increasing polarity to obtain *n*-hexane (H), methylene chloride (MC), EtOAc (E), *n*-butanol (B) fractions, and water-soluble residue (DW), respectively.

The antioxidant activities of fractions against radicals were obtained as follows:

%EC = (A control − A sample) × 100/(A control)

A sample = absorbance of the sample, A control = absorbance of untreated sample.

Results were express as EC_50_, which correspond to samples (μg/mL) required to inhibition at 50% of the initial DPPH^•^ and ABTS^•+^ radicals under the given experimental conditions.

### 2.4. Fractionation and Separation of Marker Compounds ***1***–***7***

Seven marker compounds were isolated as follows: dried leaves (4.68 kg) of *V. rotundifolia* were extracted with 80% EtOH by sonification for 90 min at three times. The soluble residue was concentrated under vacuum to obtain 1.30 kg of total extract. This total extract was then suspended in water and was partitioned with n-hexane, methylene chloride, EtOAc, *n*-butanol solvents to get H (147.8 g), MC (37.3 g), E (148.7 g), B (393 g) fractions, and water residue (450 g), respectively.

Subsequently, the E fraction was chromatographed on silica gel column chromatography eluting with a gradient solvent system of methanol in MC from 10 to 100% buffered with 0.01% of water to obtain nine fractions (E1−E9). Fraction E2 was subjected to a prep-HPLC using Triart C_18_ (10 × 250 mm, 5 μm, YMC, Tokyo, Japan; detected at wavelength 254 nm; flow rate 3.0 mL/min) column eluting with a mobile phase of water (containing 0.3% formic acid, A) and acetonitrile (B) as a gradient solvent system from 0 min (6% B) to 90 min (25% B) to obtain compounds **1** (t_R_ 46.0 min) and **3** (t_R_ 60.0 min). Fraction E4 was loaded to the above separation condition using a gradient solvent system from 0 min (14% B) to 90 min (35% B) to obtain compound **6** (t_R_ 38.5 min). Fraction E4 was separated using the above prep-HPLC Triart C_18_ (10 × 250 mm, 5 μm, YMC, Tokyo, Japan; detected at wavelength 254 nm; flow rate 3.0 mL/min) using a gradient solvent system from 0 min (15% B) to 55 min (23% B) to obtain **5** (t_R_ 34.0 min) and **7** (t_R_ 46.5 min). Compound **2** (t_R_ 38.5 min) was isolated from aqueous residue fraction by the above prep-HPLC using Triart C_18_ (20 × 250 mm, 5 μm, YMC, Tokyo, Japan; detected at wavelength 254 nm; flow rate 3.0 mL/min) condition eluting with a gradient solvent system from 0 min (13% B) to 65 min (40% B). Compound **4** (t_R_ 31.7 min) was isolated from n-hexane fraction by the above prep-HPLC condition using Triart C_18_ (20 × 25 mm, 5 μm, YMC, Tokyo, Japan; detected at wavelength 254 nm; flow rate 3.0 mL/min) by an isocratic elution of 15% (B) from 0 to 50 min.

### 2.5. Chromatographic and Separation Conditions

The chemical profiling of five organs of *V. rotundifolia* together with quantification and validation of seven marker compounds were experimented on an HPLC chromatography (Waters, Houston, TX, USA) equipped with a photodiode array (PDA) detector at 25 °C. The HPLC components were conducted by using an auto-sampler, degasser, and quaternary solvent pump for quantitative analysis. The seven marker compounds and samples were conducted by using an HPLC C18 column (4.6 × 250 mm, 5 µm particle size; YMC, Tokyo, Japan) at 35 °C; flow rate of 0.8 mL/min; injection volume of 10 μL. The detection was performed with an ultra-violet (UV) detector at wavelength of 254 and 326 nm. The mobile phase consisted of a solvent system of phase A (water containing 0.1% formic acid) and phase B (CH3CN) with gradient elution as follows: 2–35% (B) from 0 to 30 min, 35–100% (B) for 3 min, 100–100% (B) for 2 min, 100–2% (B) for 2 min, and held for 8 min. The column was then re-equilibrated with 2% (B) until the end of analysis.

### 2.6. Method Validation

#### 2.6.1. Detection of Wavelength

**Figure 1:**
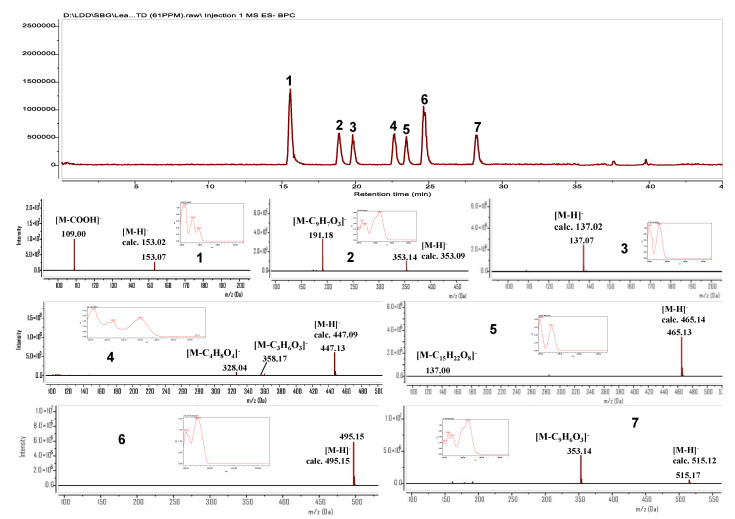
UV- and MS spectra of marker compounds (**1**–**7**).

Seven marker compounds were firstly checked for their purities, ranging from 96.65% to 98.46%. Compounds **1**, **3**, **5**, and **6**, phenolics, showed the UV absorption maxima at 196, 218, 248, 254, and 259 nm. Compound **4**, flavonoid glycoside, showed the UV absorption maxima at 219, 269, and 349 nm. Compounds **2** and **7**, caffeoylquinic acids (CQAs), displayed the UV absorption maxima at 326 nm. Therefore, the UV wavelengths were collected at 254 and 326 nm for detecting four compounds (**1**, **3**, **5**, and **6**) and three compounds (**2**, **4,** and **7**), respectively (Figure 1).

#### 2.6.2. Preparation of Calibration Standard Solution

Seven marker compounds **1**–**7** reached over 96.65% of purities based on the detection of their signals on HPLC-PDA system. Standard stock was prepared at a concentration of 1000 μg/mL accurately, and then was diluted by addition with a volume of MeOH to approach the working concentrations. These standard solutions were kept in brown glass vials of 5 mL filmed by plastic film (Parafilm, Chicago, IL, USA) and were stored in a refrigerator at 4 °C. The calibration curves were built using five different concentrations for each analyte. In detail, the concentrations were prepared ranging from 0.98 to 125 μg/mL for marker compounds **1**–**3**, 12.5–1600 μg/mL for compound **4**, 0.98–125 μg/mL for compound **5**, 0.95–62.5 μg/mL for compound **6**, and 0.95–31.25 μg/mL for compound **7**. The linearity was conducted for calibration curves at triplicated time in an independent manner, and limit of detection (LOD) and limit of quantification (LOQ) values were obtained (Table S1, Supplementary Materials).

### 2.7. Mass Analysis

The mass confirmation of compounds **1**–**7** was carried out by HPLC chromatography (Waters, Houston, TX, USA) coupled with a Waters Quattro Micro Mass™ (Waters, Milford, MA, USA) equipped with an electrospray ionization (ESI) source. The instrument was operated in positive and negative ions mode. MS conditions were as follows: capillary voltage, 3.0 kV; extractor voltage, 3 V; cone voltage, 50 V; RF lens voltage, 0 V; source temperature, 100 °C; desolvation temperature, 300 °C; desolvation gas, 450 L/h; cone gas, 50 L/h; collision gas, 0.14 mL/min. The gradient elution was performed using the analytical method mentioned above.

### 2.8. Screened Antioxidants by DPPH-HPLC Analysis and Further Verified by ELISA Assay

The DPPH-HPLC method is an effective analytical method for evaluation of the radical screening activity of compound based on the reduction of the peak areas of compound interacting with blank solution and DPPH, respectively, in the HPLC chromatograms. The leaf extract showed the most abundant biomass of maker compounds. Thus, this extract was accessed for its antioxidant property using a reported method [20]. The 80% EtOH extract of *V. rotundifolia* leaves was added by DPPH solution (40 µL, 0.4 mg/mL), then this reaction mixture was incubated for 30 min at 37 °C. After that, the mixture was filtered through 0.45 μm filter for HPLC analysis. The same extract concentration was added with blank methanol of 40 µL as a control. Both of the mixtures were analyzed using the same established analytical methods. The effluent was monitored at 254 and 326 nm wavelengths.

Subsequently, the active compounds **1**, **2**, **4**, and **7** were further tested for their antioxidant activity using the above-described DPPH method on ELISA to verify their DPPH^•^ radical scavenging properties. These marker compounds were prepared at concentrations ranging from 5 to 100 μM.

### 2.9. Anti-Inflammatory Assay

The anti-inflammatory effects of the active fractions from five organs of *V. rotundifolia* were evaluated using our previous method [21].

#### 2.9.1. Cell Culture

RAW264.7 cell lines, which are mouse-derived macrophages, were purchased from the Bank of Korea, and cultured in DMEM medium containing 10% FBS, 100 U/mL penicillin, and 100 μg/mL streptomycin. Cells were cultured in a 75T flask at 37 °C in humidified atmosphere containing 5% CO_2_.

#### 2.9.2. Measurement of Cell Viability

The cytotoxicity of extracts, fractions, and compounds was determined using an MTT assay [21]. RAW264.7 cells were seeded at a concentration of 10^5^ cells/well in 96-well plates in DMEM containing 10% FBS for 24 h. Cells were treated with various concentrations of samples for 1 h before stimulation with LPS (1 µg/mL) for 16 h. Cell viability was assessed by MTT (3-[4,5-dimethyl-2-thiazolyl]-2,5-diphenyl tetrazolium bromide) assay. Cultured cells were incubated with MTT (0.05 mg/mL) at 37 °C for 4 h. The supernatants were then removed and then monitored at 570 nm in a microplate reader. Control was prepared at the same condition without treated sample.

#### 2.9.3. Measurement of Nitric Oxide (NO) Production

RAW 264.7 cells were pre-incubated for 1 h with the mentioned compounds and were subsequently stimulated with LPS (1 µg/mL). After 16 h, the supernatant was harvested and the cultured RAW264.7 cells (1 × 10^5^ cells/well) were treated with an equal amount of Griess reagent (equal volumes of 1% (*w*/*v*) sulfanilamide in 5% (*v*/*v*) phosphoric acid and 0.1% (*w*/*v*) naphtylethylene). The cultured supernatant was incubated at room temperature for 10 min, and the absorbance was measured at 550 nm using a microplate reader. Serum-free culture medium was used to measure nitrite production. The control and negative controls were prepared at the same experimental conditions in the presence or absence of LPS stimulation without sample treatment.

### 2.10. Statistical Analysis

Data were represented as the means ± standard deviations (S.D.) (*n* = 3) of three replicates. The nonparametric one-way ANOVA followed by Dunnett’s multiple comparison test using the Graphprism version 8.0.1 software (GraphPad Software, La Jolla, CA, USA) was used for statistical analyses. * *p* < 0.05, ** *p* < 0.01, compared to controls, accepted as statistically significant.

## 3. Results

### 3.1. Screen DPPH and ABTS Activities Guided Extraction Solvent Selection

DPPH assay is a simple and inexpensive method to investigate the free radical capacity of samples collected from Goheung based on a reaction between free radical DPPH^•^ and hydrogen donor absorbance at 515 nm. The amounts of free DPPH radicals were scavenged by tested samples and calculated with reference to control (without sample addition). As a result, the 80% ethanol extracts of five organs exhibited stronger radical scavenging capacity than those of 100% MeOH extracts at concentrations of 10 and 100 µg/mL (Table 1). Notably, leaf, twig, and flower extracts of *V*. *rotundifolia* exhibited significant reducing capacity on DPPH^•^ radicals (64.16%, 68.25%, and 53.46%, respectively) than those of 100% MeOH extract (70.20%, 78.15%, and 77.09%, respectively) at concentrations of 100 µg/mL. In contrast, the 80% EtOH extracts of twigs and flowers of *V*. *rotundifolia* were more effective in scavenging ABTS^•+^ radicals than those of 100% MeOH extracts.

Furthermore, obtained extract yields using 80% EtOH were higher than those using 100% MeOH. Therefore, 80% EtOH was selected as the solvent extraction for further studies. The extraction time was also investigated for 30, 60, 90, and 120 min. At the end of each period, each sample was collected and filtered. Next, each analyte contained in the sample was calculated by analysis of their peak areas corresponding to those at different extraction time using the same analytical method conditions. The results indicated that compounds **1**–**7** increased their contents from 30 to 90 min, and the same number of them obtained from 90 to 120 min (Figure S1, Supplementary Materials). Thus, the optimal condition of 90 min for extraction was set up during the experiment.

### 3.2. Antioxidant Properties of Fractions

The 80% EtOH extracts of five organs from *V*. *rotundifolia* were successfully partitioned into H, MC, E, B, and DW fractions. The antioxidant properties of these fractions were also evaluated for scavenging activities on DPPH^•^ and ABTS^•+^ radicals. In ABTS experiment, the E fraction (EC_50_ = 32.85 µg/mL) derived from the flowers and the MC fraction (EC_50_ = 35.11 µg/mL) from roots showed the potent free radical scavenging activity. In addition, the E fractions from roots (EC_50_ = 55.30 µg/mL), leaves (EC_50_ = 70.77 µg/mL), and fruits (EC_50_ = 70.98 µg/mL) displayed significant antioxidant capacities. Moreover, the B fractions from flowers, roots, twigs, fruits, and leaves, together with the E fractions from twigs as well as the MC fractions from leaves, fruits, and twigs, also exhibited moderate antioxidant activity, with EC_50_ values ranging from 104.99 to 224.12 µg/mL (Table 2). In DPPH scavenging properties, the E fraction from flowers exhibited the strong antioxidant activity (EC_50_ = 19.10 µg/mL), followed by E fractions from fruits (EC_50_ = 35.61 µg/mL), leaves (EC_50_ = 53.05 µg/mL), and roots (EC_50_ = 68.20 µg/mL). The B fraction (EC_50_ = 70.00 µg/mL) derived from flowers and the MC fraction from roots (EC_50_ = 72.86 µg/mL) showed the moderate free radical scavenging activity. The E fraction derived from the twigs exhibited significant free radical scavenging activity with an EC_50_ value of 87.12 µg/mL. In contrast, the B fractions from leaves, fruits, twigs, and roots exhibited moderate antioxidant activity with EC_50_ ranging from 124.88 to 202.30 µg/mL. The MC fraction from leaves displayed a weak activity with an EC_50_ > 250 µg/mL (Table 2).

### 3.3. Isolation and Identification of Marker Compounds ***1***–***7***

#### Spectroscopic Data of Compounds **1**–**7**

Protocatechuic acid (**1**): White amorphous powder; ESI-MS: 109.0 [M-COOH]^−^,153.1 [M-H]^−^ (C_7_H_5_O_4_); ^1^H-NMR (400 MHz, CD_3_OD): 6.82 (1H, d, J = 8.2 Hz, H-5), 7.45 (1H, d, J = 2.1 Hz, H-2), 7.48 (1H, dd, J = 2.1, 8.2 Hz, H-6); ^13^C-NMR (100 MHz, CD_3_OD): 124.0 (C-1), 117.7 (C-2), 145.8 (C-3), 151.4 (C-4), 115.8 (C-5), 122.9 (C-6), 170.4 (C-7).

Chlorogenic acid (**2**): White amorphous powder; ESI-MS: 191.2 [M-C_9_H_7_O_3_]^−^, 353.1 [M-H]^−^ (C_16_H_17_O_9_); ^1^H-NMR (400 MHz, CD_3_OD): 2.00–2.20 (4H, overlap), 3.69 (1H, m, H-4), 4.14 (1H, m, H-3), 5.31 (1H, m, H-5), 6.23 (1H, d, J = 16.0 Hz, H-β), 6.74 (1H, d, J = 8.2 Hz, H-5′), 6.91 (1H, dd, J = 2.3, 8.2 Hz, H-6′), 7.01 (1H, d, J = 2.3 Hz, H-2′), 7.52 (1H, d, J = 16.0 Hz, H-α); ^13^C-NMR (100 MHz, CD_3_OD): 76.2 (C-1), 38.2 (C-2), 71.3 (C-3), 73.5 (C-4), 72.0 (C-5), 38.8 (C-6), 177.2 (C-7), 127.8 (C-1′), 115.2 (C-2′), 146.8 (C-3′), 149.6 (C-4′), 116.5 (C-5′), 123.0 (C-6′), 147.1 (C-α), 115.3 (C-β), 168.7 (C-9′).

*4*-Hydroxybenzoic acid (**3**): White amorphous powder; ESI-MS: 137.1 [M-H]^−^ (C_7_H_5_O_3_); ^1^H-NMR (400 MHz, CD_3_OD): 6.80 (2H, d, J = 8.9 Hz, H-3, 5), 7.86 (2H, d, J = 8.9 Hz, H-2, 6); ^13^C-NMR (100 MHz, CD_3_OD): 122.6 (C-1), 133.0 (C-2, 6), 116.0 (C-3, 5), 163.2 (C-4), 170.3 (C-7).

Orientin (**4**): Yellow amorphous powder; ESI-MS: 328.0 [M-C_4_H_8_O_4_]^−^, 358.2 [M-C_3_H_6_O_3_]^−^, 447.1 [M-H]^−^ (C_21_H_19_O_11_); ^1^H-NMR (400 MHz, DMSO-d_6_): 3.10–3.20 (overlap, H-2, 4), 3.39 (1H, dd, J = 6.7, 10.2 Hz, H-6″a), 3.68 (1H, dd, J = 5.4, 10.2 Hz, H-6″b), 4.48 (1H, t, J = 5.9 Hz, H-5″), 4.58 (1H, d, J = 9.7 Hz, H-1″), 4.62 (1H, m, H-3″), 6.48 (1H, s, H-3), 6.67 (1H, s, H-6), 6.89 (1H, d, J = 8.2 Hz, H-5′), 7.39 (1H, d, J = 2.2 Hz, H-2′), 7.42 (1H, dd, J = 2.2, 8.2 Hz, H-6″); ^13^C-NMR (100 MHz, DMSO-d_6_): 160.8 (C-2), 103.5 (C-3), 182.0 (C-4), 163.3 (C-5), 93.6 (C-6), 163.7 (C-7), 102.9 (C-8), 156.3 (C-9), 108.9 (C-10), 121.5 (C-1′), 113.4 (C-2′), 145.8 (C-3′), 149.8 (C-4′), 116.1 (C-5′), 119.1 (C-6′), 73.1 (C-1″), 70.7 (C-2″), 79.0 (C-3″), 70.3 (C-4″), 81.7 (C-5″), 61.6 (C-6″).

Agnuside (**5**): Amorphous powder; ESI-MS: 137.0 [M-C_15_H_22_O_8_]^-^, 465.1 [M-H]^−^ (C_22_H_25_O_11_); ^1^H-NMR (400 MHz, CD_3_OD): 2.70 (1H, m, H-5), 2.99 (1H, t, J = 7.5 Hz, H-9), 3.24 (1H, m, H-2′), 3.30 (1H, m, H-4′), 3.32 (1H, m, H-3′), 3.40 (1H, m, H-5′), 3.65 (1H, dd, J = 5.4, 11.9 Hz, H-6′a), 3.85 (1H, m, H-6′b), 4.70 (1H, d, J = 7.8 Hz, H-1′), 4.47 (1H, m, H-6), 4.90 (overlap, H-10a), 5.00 (1H, d, J = 7.4 Hz, H-1), 5.07 (1H, d, J = 14.0 Hz, H-10b), 5.12 (1H, dd, J = 4.0, 6.1 Hz, H-4), 5.83 (1H, brs, H-7), 6.34 (1H, dd, J = 1.9, 6.1 Hz, H-3), 6.84 (2H, d, J = 8.7 Hz, H-3″, 5″), 7.92 (2H, d, J = 8.7 Hz, H-2″, 6″). ^13^C-NMR (100 MHz, CD_3_OD): 97.9 (C-1), 141.8 (C-3), 105.5 (C-4), 46.3 (C-5), 82.9 (C-6), 132.4 (C-7), 142.9 (C-8), 48.6 (C-9), 63.7 (C-10), 100.2 (C-1′), 74.9 (C-2′), 78.3 (C-3′), 71.5 (C-4′), 78.0 (C-5′), 62.7 (C-6′), 122.1 (C-1″), 132.9 (C-2″, 6″), 116.2 (C-3″, 5″), 163.7 (C-4″), 167.8 (C-7″).

6′-p-Hydroxybenzoylmussaenosidic acid (**6**): Amorphous powder; ESI-MS: 495.1 [M-H]^−^ (C_23_H_27_O_12_); ^1^H-NMR (400 MHz, CD_3_OD): 1.24 (3H, s), 1.30 (1H, m, H-6a), 1.59 (2H, m, H_2_-7), 2.08 (1H, m, H-9), 2.24 (1H, m, H-6b), 3.14 (1H, m, H-5), 3.24 (1H, d, J = 8.7 Hz, H-2′), 3.42 (overlap, H-3′, 4′), 3.59 (1H, m, H-5′), 4.43 (1H, dd, J = 6.1, 11.9 Hz, H-6′a), 4.62 (1H, dd, J = 2.5, 11.9 Hz, H-6′b), 4.73 (1H, d, J = 7.8 Hz, H-1′), 5.17 (1H, d, J = 6.1 Hz, H-1), 6.81 (2H, d, J = 8.8 Hz, H-3″, 5″), 7.31 (1H, brs, H-3), 7.88 (2H, d, J = 8.8 Hz, H-2″, 6″); ^13^C-NMR (100 MHz, CD_3_OD): 95.6 (C-1), 150.2 (C-3), 111.9 (C-4), 33.4 (C-5), 30.9 (C-6), 39.8 (C-7), 81.1 (C-8), 52.1 (C-9), 25.0 (C-10), 99.8 (C-1′), 74.8 (C-2′), 77.9 (C-3′), 71.8 (C-4′), 75.7 (C-5′), 64.3 (C-6′), 122.2 (C-1″), 132.9 (C-2″, 6″), 116.2 (C-3″, 5″), 163.7 (C-4″), 167.9 (C-7″).

3,5-Di-CQA (**7**): White amorphous powder; ESI-MS: 353.1 [M-C_9_H_6_O_3_]^−^, 515.1 [M-H]^−^ (C_25_H_23_O_12_); ^1^H-NMR (400 MHz, CD_3_OD): 2.16 (1H, dd, J = 6.6, 14.0 Hz, H-2a), 2.20 (2H, m, H-6), 2.34 (1H, dd, J = 4.3, 13.5 Hz, H-2b), 3.98 (1H, dd, J = 3.4, 7.6 Hz, H-4), 5.39 (1H, m, H-5), 5.44 (1H, m, H-3), 6.27 (1H, d, J = 15.5 Hz, H-β), 6.35 (1H, d, J = 16.0 Hz, H-β’), 6.78 (2H, d, J = 8.2 Hz, H-5′, 5″), 6.97 (2H, m, H-6′, 6″), 7.07 (2H, brs, H-2′, 2″), 7.58 (1H, d, J = 15.5 Hz, H-α), 7.62 (1H, d, J = 16.0 Hz, H-α’); ^13^C-NMR (100 MHz, CD_3_OD): 74.7 (C-1), 36.0 (C-2), 72.5 (C-3), 70.7 (C-4), 72.0 (C-5), 37.6 (C-6), 177.4 (C-7), 127.9 (C-1′), 115.2 (C-2′), 146.7 (C-3′), 149.5 (C-4′), 116.5 (C-5′), 123.0 (C-6′), 147.0 (C-α), 115.1 (C-β), 168.4 (C-7′), 127.8 (C-1″), 115.5 (C-2″), 146.7 (C-3″), 149.5 (C-4″), 116.5 (C-5″), 123.1 (C-6″), 147.3 (C- α’), 115.1 (C-β’), 168.9 (C-7″).

Seven marker compounds (**1**–**7**) were obtained using multiple chromatographic methods, including open column, YMC-MPLC, and prep-HPLC. Their structures were established via analysis of their spectroscopic data and comparison with those reported in the literature. In addition, the structures of compounds **1**–**7** were further verified via observation of their calculated and experimental molecular weights (Figure 1).

**Figure 2:**
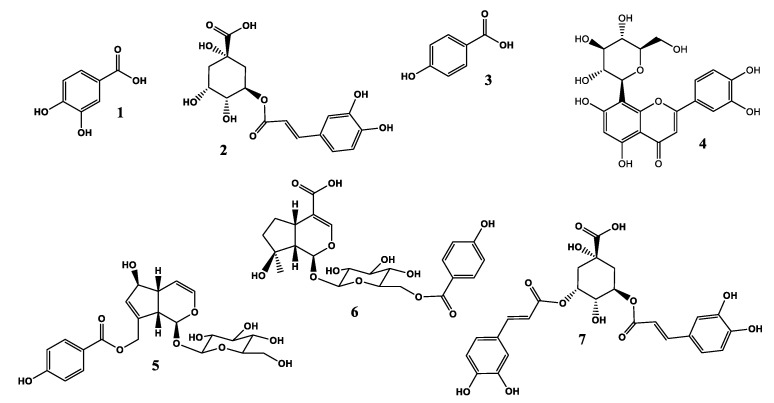
Chemical structures of marker compounds **1**–**7** isolated from the leaves of *V. rotundifolia*.

These compounds were identified as protocatechuic acid (**1**) [22], chlorogenic acid (**2**) [23], 4-hydroxybenzoic acid (**3**) [24], orientin (**4**) [25], agnuside (**5**) [26], 6′-p-hydroxybenzoylmussaenosidic acid (**6**) [27], and 3,5-di-CQA (**7**) [23] (Figure 2).

### 3.4. Establishment, Chemometric Profile, and Validation of HPLC/MS Analytical Method

#### 3.4.1. Establishment of Analytical Method

The current analytical method was based on a previous method [28] with some modification. The chromatographic profiles of five organs were obtained by optimization of analytical factors including mobile phase, gradient elution, flow rate, column, and wavelength detection to achieve the optimal resolution for separation. Many mobile phases systems were experimented using methanol, acetonitrile, and aqueous solvents containing different buffer solutions with and without chemical reagents such as formic acid, acetic acid, and phosphoric acid to enhance the resolution, restrain the ionization as well as reduce tailing of the peak detection. Formic acid was the most effective buffer in the aqueous phase. The mobile phase, including channel A (pure water containing 0.1% formic acid) and channel B (grade acetonitrile), yielded the best symmetric peaks and resolution of peak separation in the chromatograms. The column temperature was set at 35 °C to ensure precision.

As a result, the UV detection of wavelengths were also selected at 254 and 326 nm during experiments. Finally, the HPLC analytical method was successfully established.

#### 3.4.2. Chemical Profiles of Five Organs of *V. rotundifolia*

**Figure 3:**
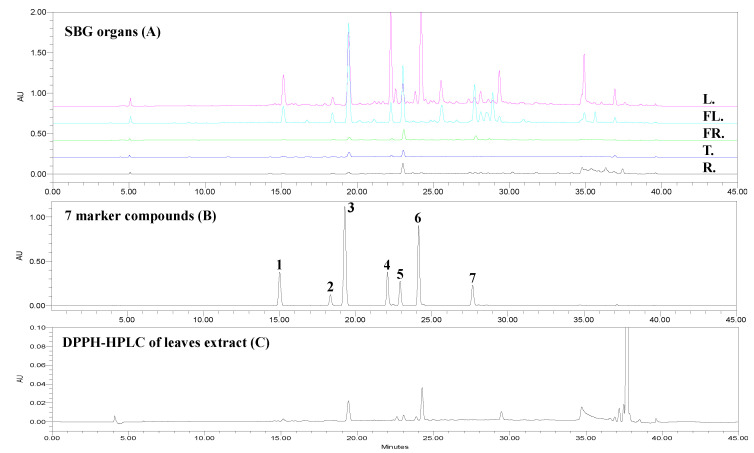
Chromatograms detected at 254 nm of five organs [(**A**), leaves (**L.**), flowers (**FL.**), fruits (**FR.**), twigs (**T.**), roots (**R.**)], seven marker compounds (**B**), and DPPH-HPLC (**C**). Seven marker compounds: protocatechuic acid (**1**), chlorogenic acid (**2**), 4-hydroxybenzoic acid (**3**), orientin (**4**), agnuside (**5**), 6′-p-hydroxybenzoylmussaenosidic acid (**6**), and 3,5-di-CQA (**7**).

The above-established method was used to analyze the chromatographic fingerprints of samples from *V*. *rotundifolia*. All the samples were filtered prior to HPLC injection. Most of the peaks were observed with a sufficiently large number of detectable signals and detected with a symmetric shape and high resolution in the chromatograms. Additionally, the signals clearly expanded in the chromatographic profiles of the five organ samples (Figure 3).

#### 3.4.3. Validation and Quantification of Marker Compounds from Organs of *V. rotundifolia*

As shown in Figure 3, most of the marker compounds showed a high resolution of separation. The presence of marker compounds in the samples were evaluated relative to their retention time and UV absorption maxima as well as MS data compared with standard mixtures in the same analytical conditions. The profiles require the relative retention times and peak areas to ensure the accuracy of data in the fingerprint analysis. Subsequently, the established method was used to validate the chemical profiling and to quantify seven marker compounds from the total extracts of five organs belonging to *V*. *rotundifolia*.

##### Linearity, LODs, and LOQs

Linearity equations were obtained with precision coefficients (*r*^2^) ranging from 0.9982 to 0.9995. LOD and LOQ values ranged from 0.15 to 0.37 and from 0.74 to 1.29 μg/mL, respectively (Table S1, Supplementary Materials). These parameters indicated the sensitivity of the analytical method.

The above-established method was used for quantification of seven marker compounds in the five organs of *V*. *rotundifolia* collected from different sampling locations.

As shown in Table 3, the content of marker compounds in each organ extract varies with the location of collection. Briefly, compounds **1** and **4** in Goheung samples were found in fruits, flowers, leaves, and twigs, but not detected in the roots. Other compounds were found in all five organs at different levels. Compound **1**, protocatechuic acid, showed the highest amount (0.4546% *w*/*w*) in the leaves, followed by 0.2446% (*w*/*w*) in flowers, 0.0040% (*w*/*w*) in fruits, and 0.0012% (*w*/*w*) in twigs. Compound **2**, chlorogenic acid, was the highest content in flowers (0.4216% *w*/*w*), followed by leaves (0.2871% *w*/*w*), fruits (0.056% *w*/*w*), roots (0.0287% *w*/*w*), and twigs (0.0271% *w*/*w*). Compound **3**, 4-hydroxybenzoic acid, exhibited the highest amount (0.4963%, *w*/*w*) in flowers, followed by leaves (0.3552% *w*/*w*), fruits (0.0184% *w*/*w*), twigs (0.0077%), and roots (0.0027%). Notably, compound **4**, orientin, was the greatest content in leaves (4.2427% *w*/*w*), followed by flowers (0.8751% *w*/*w*), fruits (0.0425% *w*/*w*), and twigs (0.0270% *w*/*w*). Compound **5**, agnuside, was the highest amount in fruits (1.4030% *w*/*w*), followed by flowers (1.1459% *w*/*w*), leaves (0.3944% *w*/*w*), and twigs and roots. Compound **6**, 6′-p-hydroxybenzoylmussaenosidic acid, was found in leaves (1.0858% *w*/*w*), followed by roots, flowers, fruits, and twigs. Significantly, compound **7**, 3,5-di-CQA, occurred at the highest level in flowers (0.6787% *w*/*w*), followed by leaves (0.0943% *w*/*w*), fruits and twigs, and roots (0.0237% *w*/*w*). The content of compound **1** in Jeju samples was the highest in leaves (0.1983% *w*/*w*), followed by flowers, twigs, and fruits (range, 0.1141–0.0039% *w*/*w*). The leaves contained the highest amount of compound **2** (2.2644% *w*/*w*) and compound **4** (1.2529% *w*/*w*), followed by flowers, fruits, and twigs. Compound **5** was the least in fruits (0.0984%), and occurred at higher levels in twigs, leaves, and flowers at 0.2011%, 0.5012%, and 0.7146% (*w*/*w*), respectively. By contrast, compound **7** was the highest (1.1429%) in the flowers, followed by leaves, twigs, and fruits.

The amounts of compounds **1**, **2**, **4**, **5**, and **6** in the leaves of Sinan samples were 0.0239%, 1.4998%, 1.0546%, 1.8050%, and 0.4516% (*w*/*w*), respectively, and the compounds were detected at lower levels in fruits, roots, and twigs. In contrast, compound **7** constituted 0.4922% (*w*/*w*) in the leaves and was detected at lower levels in twigs, roots, and fruits.

Among Busan samples, the proportion of compounds **1** and **3** in the fruits was 0.0146% and 0.0282% (*w*/*w*), respectively, compared with compounds **2** and **7** constituting 0.8450% and 0.7905% (*w*/*w*), respectively, and further reduced in the leaves, fruits, and roots. Compound **4** constituted 1.2095% (*w*/*w*) of the marker content in the leaves, followed by fruits, twigs, and roots.

Therefore, the differences in the content of marker compounds may be useful to evaluate the quality of each organ of this plant. In addition, the variation in the composition of the same organs also reflects the specific sampling location of the plant. Notably, the quantification of marker compounds of organs obtained from different locations also determined the selection of materials for further investigation.

### 3.5. Screen Antioxidants by DPPH-HPLC Method and ELISA Assay

The DPPH-HPLC is a novel strategy for rapid screening and identification of free radical scavenging activity of constituents derived from natural sources [20]. The above-established method was used to determine the antioxidant capacity of constituents derived from *V*. *rotundifolia* leaves based on evaluation of reduced peak areas between treated and untreated samples under the same experimental conditions. In contrast, the MeOH-treated sample showed 100% peak area. The result indicated that compounds **4** and **7** exhibited strong antioxidant capacity with the reduction of peak areas of 84.8% and 89.1%, respectively. Compounds **1** and **2** expressed significant antioxidant activity with a reduction of 69.54% and 77.39%, respectively. Other compounds showed a weak antioxidant effect with a small reduction in peak areas.

Subsequently, the DPPH free radical scavenging activity of the most active marker compounds was further verified using ELISA. As a result, compound **7** showed the most effective antioxidant capacity with the lowest EC_50_ value of 25.05 μM, stronger than the positive control (ascorbic acid, EC_50_ = 46.04 μM), followed by significant antioxidant activity of compounds **1** and **4** with EC_50_ values of 52.15 and 68.25 μM, respectively (Table 4).

### 3.6. Anti-Inflammatory Effects of Extracts and Fractions Derived from Five Organs

The anti-inflammatory effects of active extracts and fractions were further evaluated against NO production in LPS-stimulated RAW264.7 cells. Among five organs, the flower extract showed the strongest inhibitory effect against NO production with an IC_50_ value of 75.06 µg/mL, followed by 100.75 µg/mL (fruit extract), 101.97 µg/mL (twig extract), 138.50 µg/mL (root extract), and 169.14 µg/mL (leaf extract) (Table 5).

By contrast, none of the total extracts had a significant effect on cell viability (Figure S18, Supplementary Materials).

Furthermore, the active antioxidant fractions were also tested for their anti-inflammatory effect. The H and MC fractions of leaf extract showed potent NO inhibition with IC_50_ values of 2.21 and 6.32 µg/mL, respectively. The E fractions (fruit and root extracts) and the B fraction (twig extract) exhibited strong inhibition against NO production with IC_50_ values of 36.24, 38.85, and 42.89 µg/mL, respectively, while the B fraction (flower extract) and E fractions (flower and twig extracts) displayed a significant inhibition against NO production with IC_50_ values of 84.42, 90.10, and 92.31 µg/mL, respectively. Other fractions showed a moderate-to-weak inhibitory effect against NO production in LPS-stimulated RAW264.7 cells.

Notably, the H and MC fractions of the leaf extract displayed significant cytotoxic effects in MTT assay at concentrations of 100 μg/mL (Figure S19, Supplementary Materials) and other fractions showed non-cytotoxic effects under the same experimental conditions at concentrations of 10, 100, 200, and 300 µg/mL (Figures S19 and S20, Supplementary Materials).

Thus, the cellular metabolic activity of these cytotoxic fractions should be investigated in further experiments to determine the antiproliferative or cytotoxic effects of these fractions.

## 4. Discussion

*V*. *rotundifolia* is used in traditional herbal medicine. It is found in the Mediterranean regions and along the seacoast in Asia. Its fruits were traditionally employed as a folk medicine to treat eye pain, headache, cold, chronic bronchitis, bacterial dysentery, and diarrhea [9,10]. Other studies also reported pharmacological activities of the herbal extract and ingredients, including antioxidant, anticancer, and cardiovascular effects in hypercholesterolemia [29,30]. Our study not only provides an overview of all the organs (fruit, flow-er, leaf, twig, and root) of this plant, but also presents sufficient evidence based on experimental results. Evaluation of plants in traditional medicine has led to the choice of *V*. *rotundifolia* for further investigation. In particular, an extraction method was established based on the antioxidant effects observed experimentally. This extraction method also meets the safety requirements for the development of preparations using safe extraction solvent, such as 80% EtOH. In order to identify the fractions associated with robust activity, the free radical scavenging activity of the fractions from each extract were evaluated.

Following successful analysis of DPPH radical scavenging activity, an analytical method was established based on the key components of seven marker compounds **1**–**7** isolated from the leaves of this plant. This analytical method has been adapted to optimize the analytical factors with strong resolution and efficiency. The method was also employed to screen the antioxidant properties of constituents derived from the leaf extract via peak areas reduction using a screening DPPH-HPLC method. Compound **7** showed a high reduction (89.06%) in peak area compared with those of untreated DPPH sample. In contrast, compounds **1**, **2**, and **4** exhibited strong DPPH radical scavenging capacities with peak areas reduction ranging from 69.54% to 84.80%. These active compounds were verified using ELISA. As shown in Table 4, compound **7** strongly reduced the peak area attribute to the most antioxidant potential with the lowest EC_50_ value of 25.1 ± 0.49 μM. Compound **4** displayed a strong peak area in screening DPPH-HPLC, and significantly expressed antioxidant activity in ELISA assay with an EC_50_ value of 45.6 ± 0.07 μM. By contrast, compounds **1** and **2** displayed significant free radical scavenging activity with EC_50_ values of 52.1 ± 0.49 and 68.3 ± 0.78 μM, associated with peak areas reduction of 69.54% and 77.39%, respectively. The ELISA data established the correlation between both antioxidant assays and indicated the reliability of the rapid screening DPPH-HPLC method. The study results were in good agreement with the antioxidant effects of active compounds reported in the literature.

Previously, protocatechuic acid (**1**) also showed potential antioxidant effects against DPPH, ABTS, Fe^3+^, and Cu^2+^, and superoxide anion radical scavenging, hydroxyl radical-scavenging, in addition to chelating effects against Fe^3+^ and Cu^2+^ in in vitro assays by scavenging free radicals mediated via donating hydrogen atom or electron and chelating metal transition ions, respectively [31]. In addition, this compound also exhibited the potential inhibition against inflammatory cytokines on LPS induced lung injury in mice [32]. Thus, this compound was considered as a natural antioxidant for use in pharmacological or food industry. Chlorogenic acid (**2**) and its derivative (3,5-di-CQA, **7**) occur widely in vegetables, fruits and herbal medicines that possess strong antioxidant activity with potential health benefits [33]. The antioxidant capacity of chlorogenic acid (**2**) may be explained based on its synthesis. In brief, this compound may donate hydrogen atoms for subsequent oxidation to respective phenoxyl radicals, followed by rapid resonance stabilization. As a result, this interaction reduces the free radicals and inhibits the oxidation reactions. Notably, chlorogenic acid showed a protective effect from Con A-induced hepatitis in mice through inhibition of Toll-like receptor 4 signaling, alleviation of infiltration, and reduction of pro-inflammatory cytokines production [34], while 3,5-di-CQA exhibited the potent chondroprotective and anti-nociceptive activities in an animal model, suggesting that this compound affected osteoarthritis based on its strong anti-inflammatory and antioxidant potentials [35]. Another study indicated that orientin (**4**) and 3,5-di-CQA (**7**) also showed potential antioxidant and DPPH radical scavenging activities [25,36]. In contrast, orientin inhibited the hyperpermeability, adhesion, and migration of leukocytes which led to protection of the vascular barrier integrity. Thereby, orientin was proposed for its ability to the treatment of vascular inflammatory diseases [37]. This compound exhibited a significant suspension in radiation-induced lipid peroxidation in mice model, supported by forming less reactive aryloxyl radicals with free radicals. The presence of C-8 glycosylation [38], together its phenolic backbone having a catechol conjugated at C-2 with free hydroxyl groups at C-3′ and C-4′, promotes the radical scavenging of this compound [39].

A structure–activity relationship was determined based on the activity of seven marker compounds. Protocatechuic acid (**1**) and 4-hydroxybenzoic acid (**3**) are hydroxybenzoic acids. However, compound **1** showed a significant antioxidant activity with a peak area reduction of 69.54% compared with 3.22% for compound **3**, suggesting that the number of hydroxyl group and its ortho position on benzene skeleton determine their antioxidant property, which may be explained in terms of hydrogen donating capacity affecting the radicals generated in the system. Similarly, chlorogenic acid (**2**) and 3,5-di-CQA (**7**) are CQA compounds. By contrast, compound **7** showed a stronger antioxidant effect than compound **2** and higher antioxidant activity than ascorbic acid (positive control), suggesting that the presence of one more caffeoyl moieties contributed to the free radicals scavenging ability. Because compound **7** is a di-CQA, it may act as the free radical scavenger, leading to the breakage of radical chain sequences with both hydrogen atom and electron transfer pathways. In contrast, compound **2** mediated the hydrogen transfer through formation of radical adduct to generate stable CQA derived radicals in an acidic or neutral environment [40].

Furthermore, the established analytical method was used to quantify these marker compounds in five organs. Interestingly, the bioactivity results suggested that the antioxidant properties of extracts derived from five organs correspond to the content of active constituents (**1**, **2**, **4**, and **7**). Briefly, the flower extract carrying the highest amounts of chlorogenic acid (**2**, 0.4216% *w*/*w*) and 3,5-di-CQA (**7**, 0.6787% *w*/*w*), and the medium level of protocatechuic acid (**1**, 0.2446% *w*/*w*), also expressed the strongest DPPH free radicals scavenging activity. The leaf extract contained high levels of protocatechuic acid (**1**, 0.4546% *w*/*w*) and orientin (**4**, 4.2427% *w*/*w*), and medium levels of chlorogenic acid (**2**, 0.2871% *w*/*w*) and 3,5-di-CQA (**7**, 0.0943% *w*/*w*) having a strong antioxidant effect. However, the content of 3,5-di-CQA in the twig extract (0.0079% *w*/*w*) was similar to that of fruit extract (0.0072% *w*/*w*). The amount of chlorogenic acid (0.0271% *w*/*w*) in twig extract was higher than that in fruit extract (0.0056% *w*/*w*). However, the twig extract showed a stronger antioxidant effect in terms of DPPH radical scavenging (68.25% *w*/*w*) than those of fruit extract (78.43% *w*/*w*) at the same concentration of 100 µg/mL. This result revealed that chlorogenic acid content affected to the antioxidant property of twig and fruit extracts. The root extract containing chlorogenic acid (**2**, 0.0287% *w*/*w*) and 3,5-di-CQA (**7**, 0.0237% *w*/*w*) showed a similar antioxidant effect compared to fruit extract. Therefore, the content of 3,5-di-CQA and chlorogenic acid (the main component responsible for the antioxidant capacity) in the total extracts may be important in determining their DPPH^•^ radical scavenging activity.

The levels of marker compounds in the organ extracts supported the creation of a database for evaluating the quality of organs and sampling locations. Our study indicated the standards and active constituents from each organ collected from different locations, which is important in selecting valuable materials for research and development of functional products from this plant. Based on the levels of constituents exhibiting robust antioxidant activity (compounds **1**, **2**, **4**, and **7**) in this study, it is possible to select the sampling location as well as the organs that meet the criteria for oriented products.

However, oxidative stress and cell damage play a role in the pathophysiology of chronic inflammatory and degenerative disorders, leading to health defects and increased incidence of chronic diseases, including diabetes, cancer, and metabolic, cardiovascular, pulmonary, and neurological disorders. The oxidative stress increases pro-inflammatory cytokines and mediators [41]. By contrast, oxidative stress leads to an imbalance in reactive oxygen species such as free radicals, reactive metabolites, and oxidants, which may be restored by protective mechanisms [28]. The strong antioxidant effects of extracts and fractions also displayed the significant inhibitory effect against NO production in LPS-stimulated RAW264.7 cells. The results also establish a relationship between antioxidant and anti-inflammatory activities. Thus, the active fractions represent interesting materials for further analysis and discovery of active compounds. In addition, the H and MC fractions potently affected cell viability in the MTT assay. These fractions are good materials for separation and identification of cytotoxic compounds, suggesting the need for further investigation into their mechanism of action. The results revealed that these fractions exhibit low cytotoxicity and represent anti-proliferative products. Further studies of these fractions are necessary to identify the active constituents for product development.

## 5. Conclusions

A bioactivity-guided experiment was used to establish effective analytical and rapid screening DPPH-HPLC methods. Extraction and separation skills were conducted to obtain seven marker compounds using multiple chromatographic methods. The structures were identified by spectroscopic analysis and further verified using UV and MS spectra. The established method was successfully employed to quantify the levels of marker compounds from five organs (fruits, flowers, leaves, twigs, and roots) of *V*. *rotundifolia* at different locations. The variable levels of compounds in each organ of this plant and the bioactive principles in the organ extracts based on different active ingredients can be used to develop functional products. Besides, the active fractions identified in this study are important for further studies investigating the antioxidant, anti-inflammatory, or toxicological activities. Further studies are needed to investigate the mechanism of action of the active compounds. Our study provides the first overview of the phytochemical analysis of all organs of *V*. *rotundifolia* and supports a useful analytical method to determine the quality of products derived from different organs of this plant.

## Figures and Tables

**Table 1:** Total amounts and antioxidant capacities (DPPH^•^ and ABTS^•+^) of organs (leaf, flower, fruit, twig, and root) extracts.

Total Extracts			Organs				
			Leaf	Flower	Fruit	Twig	Root
**80% EtOH** (mg/g dried sample)			27.2	16.0	40.0	24.2	2.4
**100% MeOH** (mg/g dried sample)			14.8	14.0	25.6	24.0	1.6
**Radical** **activity**	**Total extract**	**Con. (μg/mL)**					
**DPPH** (%)	**80% EtOH**	10	90.28 ± 9.55 *	101.43 ± 2.60 **	94.37 ± 10.73	94.00 ± 15.47	98.09 ± 3.09 *
		100	64.16 ± 2.91	53.46 ± 3.57	78.43 ± 6.59	68.25 ± 9.42	68.25 ± 6.12
	**100% MeOH**	10	95.12 ± 0.85 *	101.97 ± 0.93	95.03 ± 5.72 *	101.49 ± 2.04 **	100.60 ± 1.49
		100	70.20 ± 2.46	77.09 ± 6.39	83.73 ± 0.49	78.15 ± 3.96	76.57 ± 1.48
	**Ascorbic acid**	10	49.15 ± 0.21 **	49.15 ± 0.21 **	49.15 ± 0.21 **	49.15 ± 0.21 **	49.15 ± 0.21 **
		100	3.91 ± 0.00 **	3.91 ± 0.00 **	3.91 ± 0.00 **	3.91 ± 0.00 **	3.91 ± 0.00 **
**ABTS**^•+^ (%)	**80% EtOH**	10	95.57 ± 0.34 *	98.76 ± 1.34 *	95.76 ± 1.98 *	94.39 ± 0.70 *	93.84 ± 0.62 **
		100	63.09 ± 1.17 *	77.18 ± 0.23 *	65.97 ± 0.47 *	58.53 ± 1.03 *	47.13 ± 0.42 **
	**100% MeOH**	10	96.76 ± 0.96 *	98.39 ± 0.72	96.03 ± 0.48 *	95.85 ± 0.28 **	93.25 ± 2.33 **
		100	65.74 ± 0.28 *	83.25 ± 1.06	67.20 ± 0.36 *	61.18 ± 0.63 **	43.52 ± 0.72 **
	**Ascorbic acid**	10	33.92 ± 0.49 **	33.92 ± 0.49 **	33.92 ± 0.49 **	33.92 ± 0.49 **	33.92 ± 0.49 **
		100	2.85 ± 0.62 **	2.85 ± 0.62 **	2.85 ± 0.62 **	2.85 ± 0.62 **	2.85 ± 0.62 **

The data are expressed as the mean ± SD (*n* = 3) of three individual experiments. Control was prepared with same conditions with the amount of sample replaced by addition of ethanol. * *p* < 0.05, ** *p* < 0.01 (Dunnett’s multiple comparisons test) compared to the control, nonparametric one-way ANOVA.

**Table 2:** Antioxidant properties of active fractions derived from five organs of *V. rotundifolia*.

Organs	Fractions	ABTS^•^+ EC_50_ Values (µg/mL)	DPPH^•^ EC_50_ Values (µg/mL)
**Leaf**	**MC**	185.48 ± 1.74	>250
	**E**	70.77 ± 0.40	53.05 ± 3.37
	**B**	224.12 ± 2.44	124.88 ± 7.30
**Flower**	**E**	32.85 ± 1.23	19.10 ± 2.94
	**B**	104.99 ± 1.64	70.00 ± 1.79
**Fruit**	**MC**	119.43 ± 1.13	>250
	**E**	70.98 ± 1.16	35.61 ± 7.64
	**B**	207.56 ± 4.76	189.17 ± 5.06
**Twig**	**E**	123.33 ± 0.95	87.12 ± 6.90
	**B**	160.75 ± 0.92	146.21 ± 16.68
**Root**	**MC**	35.11 ± 0.19	72.86 ± 2.41
	**E**	55.30 ± 0.75	68.20 ± 1.30
	**B**	124.90 ± 2.60	202.30 ± 15.26
	Ascorbic acid *	6.35 ± 3.30	8.38 ± 0.40

EC_50_ values represent the concentrations of samples having 50% of their maximal effect against DPPH/ABTS free radicals. * Positive control.

**Table 3:** Quantification of marker compounds content in the organs of *V. rotundifolia* collected at different locations.

Locations	Samples	Marker Compounds						
		1	2	3	4	5	6	7
**Goheung**	**Leaf**	0.4546 ± 0.0036	0.2871 ± 0.0008	0.3552 ± 0.0010	4.2427 ± 0.0012	0.3944 ± 0.0030	1.0858 ± 0.0001	0.0943 ± 0.0013
	**Flower**	0.2446 ± 0.0022	0.4216 ± 0.0031	0.4963 ± 0.0020	0.8751 ± 0.0023	1.1459 ± 0.0043	0.0062 ± 0.0001	0.6787 ± 0.0040
	**Fruit**	0.0040 ± 0.0001	0.0056 ± 0.0000	0.0184 ± 0.0003	0.0425 ± 0.0009	1.4030 ± 0.0010	0.0039 ± 0.0001	0.0072 ± 0.0001
	**Twig**	0.0012 ± 0.0001	0.0271 ± 0.0003	0.0077 ± 0.0002	0.0270 ± 0.0001	0.2129 ± 0.0027	0.0037 ± 0.0001	0.0079 ± 0.0003
	**Root**	N.D.	0.0287 ± 0.0001	0.0027 ±0.0001	N.D.	0.2112 ± 0.0009	0.0095 ± 0.0001	0.0237 ± 0.0003
**Jeju**	**Leaf**	0.1983 ± 0.0007	2.2644 ± 0.0001	0.1433 ± 0.0011	1.2529 ± 0.0075	0.5012 ± 0.0045	0.1109 ± 0.0001	0.7863 ± 0.0003
	**Flower**	0.1141 ± 0.0002	0.5471 ± 0.0010	0.2475 ± 0.0010	0.5781 ± 0.0034	0.7146 ± 0.0017	0.0043 ± 0.0001	1.1429 ± 0.0024
	**Fruit**	0.0039 ± 0.0001	0.1330 ± 0.0013	0.0168 ± 0.0002	0.2427 ± 0.0006	0.0984 ± 0.0007	0.0018 ± 0.0001	0.0809 ± 0.0003
	**Twig**	0.0514 ± 0.0001	0.1226 ± 0.0010	0.0396 ± 0.0002	0.0742 ± 0.0007	0.2011 ± 0.0002	0.0021 ± 0.0001	0.4464 ± 0.0008
**Sinan**	**Leaf**	0.0239 ± 0.0003	1.4998 ± 0.0067	0.0042 ± 0.0001	1.0546 ± 0.0024	1.8050 ± 0.0033	0.4516 ± 0.0014	0.4922 ± 0.0034
	**Fruit**	0.0080 ± 0.0004	0.9908 ± 0.0014	0.0182 ± 0.0002	0.6788 ± 0.0015	0.7085 ± 0.0014	0.0134 ± 0.0003	0.1416 ± 0.0002
	**Twig**	N.D.	0.1949 ± 0.0005	N.D.	N.D.	0.4229 ± 0.0008	0.0032 ± 0.0001	0.2466 ± 0.0006
	**Root**	N.D.	0.3941 ± 0.0003	0.0023 ± 0.0001	N.D.	0.5594 ± 0.0003	0.0006 ± 0.0001	0.2451 ± 0.0003
**Busan**	**Leaf**	N.D.	0.5883 ± 0.0005	0.0022 ± 0.0001	1.2095 ± 0.0038	0.7462 ± 0.0008	0.0519 ± 0.0003	0.3789 ± 0.0004
	**Fruit**	0.0146 ± 0.0004	0.2424 ± 0.0002	0.0282 ± 0.0007	0.6423 ± 0.0003	0.0067 ± 0.0001	N.D.	0.1255 ± 0.0006
	**Twig**	N.D.	0.8450 ± 0.0057	N.D.	0.1631 ± 0.0005	N.D.	N.D.	0.7905 ± 0.0042
	**Root**	N.D.	0.3527 ± 0.0011	N.D.	N.D.	0.4607 ± 0.0026	N.D.	N.D.

Values are mean ± SD (*w*/*w*: weight of compound per weight of dried materials, %) and were experimented at three times with an independent manner. N.D. not detected.

**Table 4:** Antioxidant effect of seven marker compounds on DPPH radical.

Compounds	^a^ Reduction of the Peak Area (%)	EC_50_ Values (µM)
Protocatechuic acid (**1**)	69.54 ± 2.33	52.15 ± 0.49
Chlorogenic acid (**2**)	77.39 ± 1.14	68.25 ± 0.78
4-Hydroxybenzoic acid (**3**)	3.22 ± 0.52	-
Orientin (**4**)	84.85 ± 1.07	56.45 ± 0.07
Agnuside (**5**)	21.82 ± 1.33	-
6′-*p*-Hydroxybenzoylmussaenosidic acid (**6**)	4.90 ± 0.21	-
3,5-Di-CQA (**7**)	89.06 ± 1.17	25.05 ± 0.49
Ascobic acid *	-	46.04 ± 0.67

* Positive control. ^a^ Reduction of peak areas between treated and untreated DPPH to samples in the leaves extract. Peaks area of the untreated DPPH sample was considered as 100%.

**Table 5:** Anti-inflammatory activity of organ extracts and their fractions in LPS-stimulated RAW264.7 cells.

Organs	IC_50_ Values (µg/mL)	Fractions	IC_50_ Values (µg/mL)
**Leaf**	169.14	**H**	2.21 ± 0.97
		**MC**	6.32 ± 0.83
		**E**	188.64 ± 5.91
		**B**	>300
		**DW**	>300
**Flower**	75.06	**E**	90.10 ± 3.95
		**B**	84.42 ± 4.29
**Fruit**	100.75	**E**	36.24 ± 6.48
		**B**	229.95 ± 34.76
**Twig**	101.97	**E**	92.31 ± 3.49
		**B**	42.89 ± 1.48
**Root**	138.50	**E**	38.85 ± 5.56
		**B**	285.98 ± 17.38

Nitrite concentrations of non-treated and LPS-treated controls were 0.6 ± 0.01 μM and 15.09 ± 0.40 μM, respectively.

## Data Availability

All of data is contained within the article.

## Supplementary Materials

The following supporting information can be downloaded at: https://www.mdpi.com/article/10.3390/antiox11030454/s1, Figures S1–S18, Table S1: Retention time and purity of marker compounds (**1**–**7**).
