# Supplementary material for: Analysis of Antioxidant Phytochemicals and Anti-Inflammatory Effect from Vitex rotundifolia L.f
Source: Antioxidants (Basel). 2022 Feb 24;11(3):454. doi: 10.3390/antiox11030454 (PMC8944582; doi:10.3390/antiox11030454)
Supplement: Supplementary file 1 [file antioxidants-11-00454-s001.zip › antioxidants-1563085-supplementary.pdf]

Supplementary Materials

Analysis of Antioxidant Phytochemicals and Anti-Inflammatory Effect from *Vitex rotundifolia* L.f.

DucDat Le, Sanghee Han, Jeongjun Ahn, Jayeon Yu, Chang-Kwon Kim and Mina Lee*

[a]College of Pharmacy and and Research Institute of Life and Pharmaceutical Sciences, Sunchon National University, 255 Jungangno, Suncheon 57922, Jeonnam, Korea

*Corresponding author: Mina Lee
🖂 E-mail: minalee@sunchon.ac.kr; Tel.: +82-61-750-3764; Fax: +82-61-750-3708.
College of Pharmacy and and Research Institute of Life and Pharmaceutical Sciences, Sunchon National University, 255 Jungangno, Suncheon 57922, Jeonnam, Korea

Table of Contents

1. Optimization of extraction time by sonification samples using 80% EtOH solvent 1

Figure S1. The amounts of analytes stimulation at different time 1

2. Spectroscopic data of marker compounds (1–7): 2

Figure S2. ^{1}H-NMR (400 MHz, CD_3OD) spectrum of compound **1** 2

Figure S3. ^{13}C-NMR (100 MHz, CD_3OD) spectrum of compound **1** 2

Figure S4. ^{1}H-NMR (400 MHz, CD_3OD) spectrum of compound **2** 3

Figure S5. ^{13}C-NMR (100 MHz, CD_3OD) spectrum of compound **2** 3

Figure S6. ^{1}H-NMR (400 MHz, CD_3OD) spectrum of compound **3** 4

Figure S7. ^{13}C-NMR (100 MHz, CD_3OD) spectrum of compound **3** 4

Figure S8. ^{1}H-NMR (400 MHz, CD_3OD) spectrum of compound **4** 5

Figure S9. ^{13}C-NMR (100 MHz, CD_3OD) spectrum of compound **4** 5

Figure S10. ^{1}H-NMR (400 MHz, CD_3OD) spectrum of compound **5** 6

Figure S11. ^{13}C-NMR (100 MHz, CD_3OD) spectrum of compound **5** 6

Figure S12. ^{1}H-NMR (400 MHz, CD_3OD) spectrum of compound **6** 7

Figure S13. ^{13}C-NMR (100 MHz, CD_3OD) spectrum of compound **6** 7

Figure S14. ^{1}H-NMR (400 MHz, CD_3OD) spectrum of compound **7** 8

Figure S15. ^{13}C-NMR (100 MHz, CD_3OD) spectrum of compound **7** 8

3. Validation method. 9

Table S1. Retention time and purity of marker compounds (**1–7**) 9

4. Biological activities 10

Figure S16. Cell viability of crude extracts at concentrations of 10 and 100 µg/mL on RAW264.7 cells. 10

Figure S17. Cell viability of crude extracts and fractions at concentrations of 10 and 100 µg/mL on RAW264.7 cells. 10

Figure S18. Cell viability of crude extracts and fractions at concentrations of 200 and 300 µg/mL on RAW264.7 cells. 11

1. Optimization of extraction time by sonification samples using 80% EtOH solvent

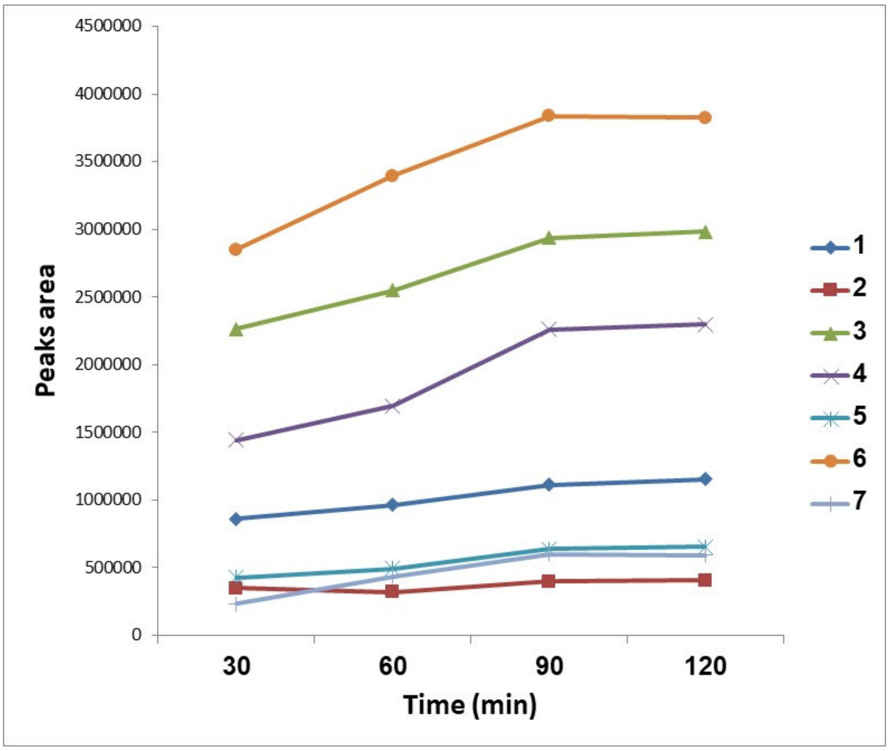

Figure S1. The amounts of analytes stimulation at different time.

2. Spectroscopic data of marker compounds (1–7):

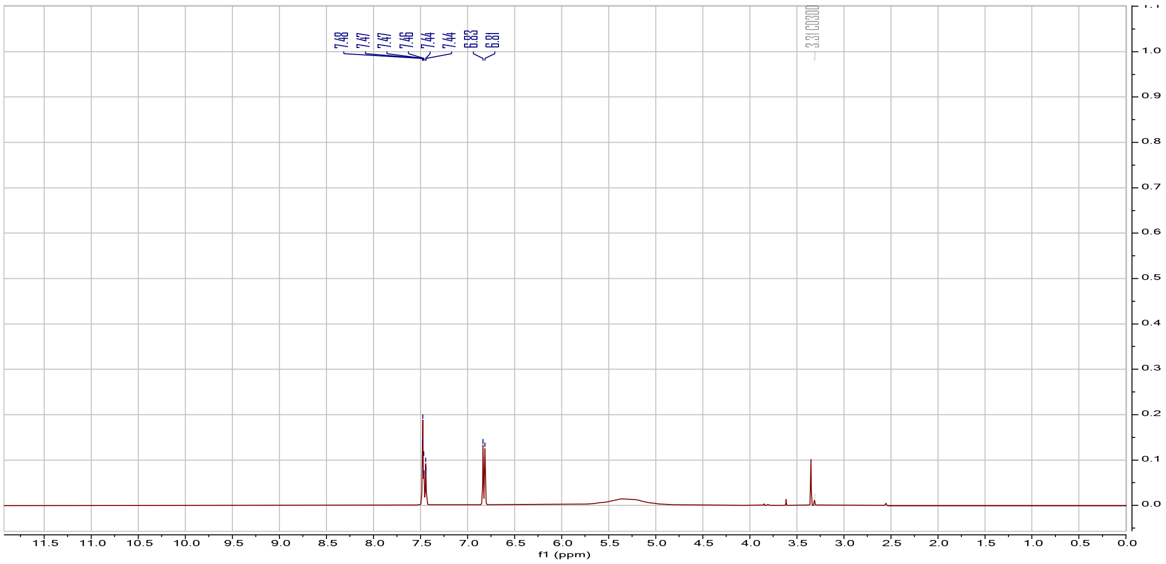

Figure S2. ^{1}H-NMR (400 MHz, CD_3OD) spectrum of compound **1**.

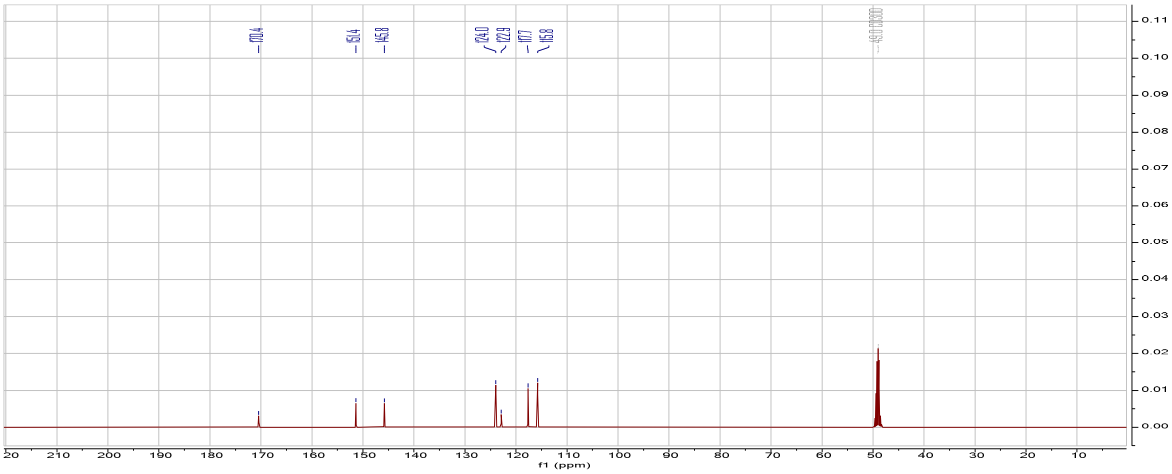

Figure S3. ^{13}C-NMR (100 MHz, CD_3OD) spectrum of compound **1**.

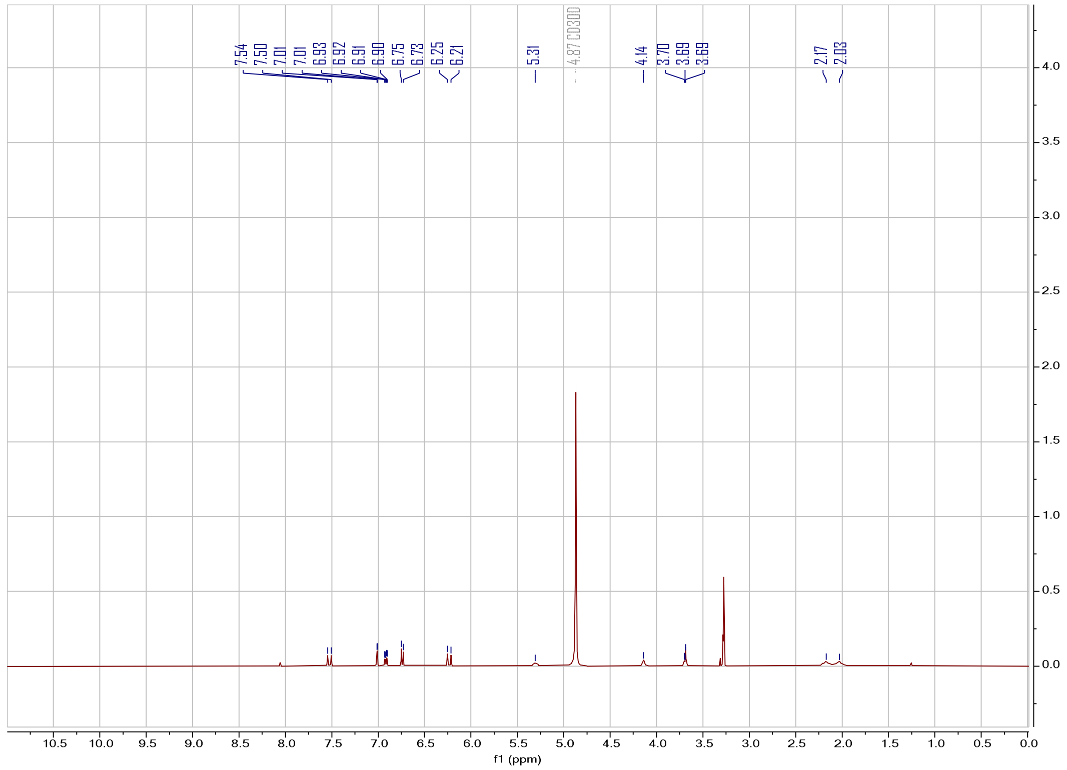

Figure S4. ^{1}H-NMR (400 MHz, CD_3OD) spectrum of compound **2**.

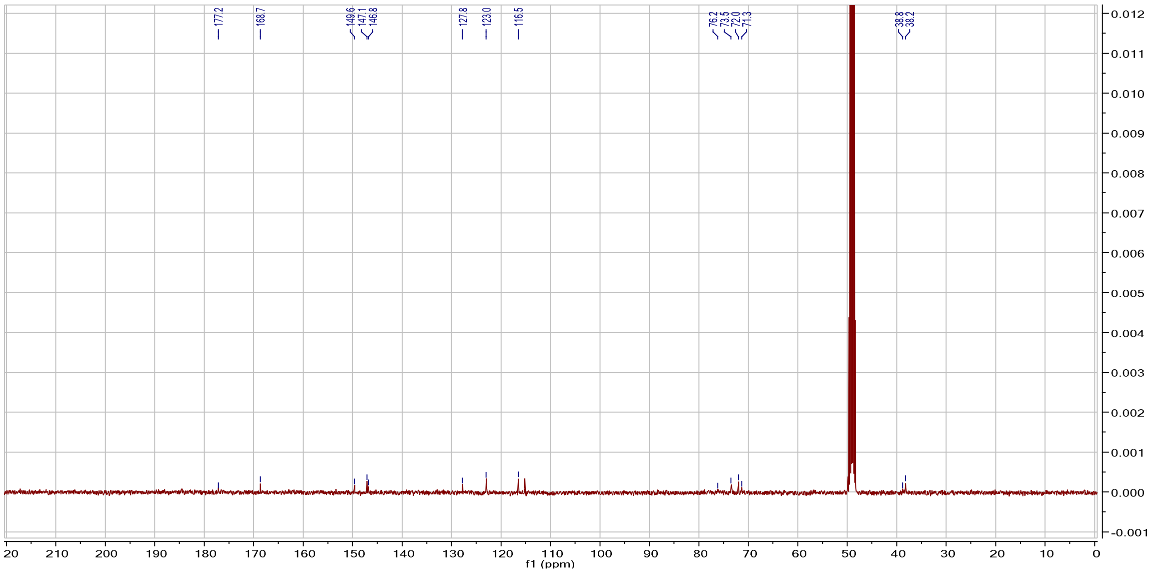

Figure S5. ^{13}C-NMR (100 MHz, CD_3OD) spectrum of compound **2**.

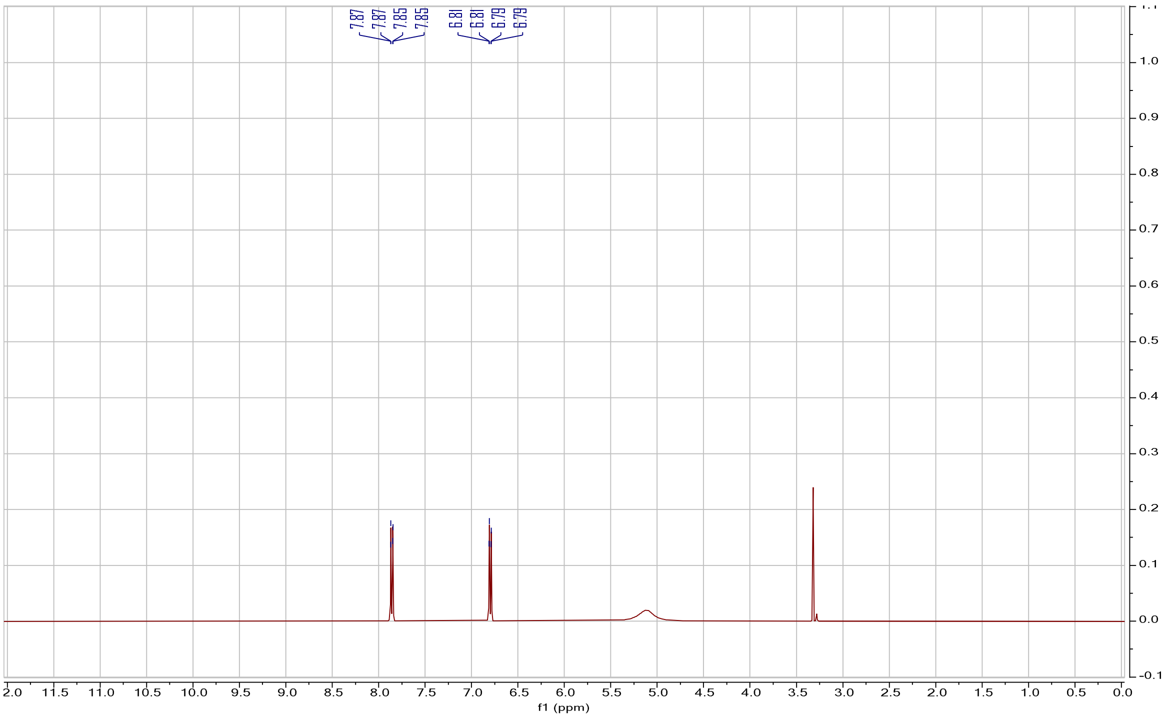

Figure S6. ^{1}H-NMR (400 MHz, CD_3OD) spectrum of compound **3**.

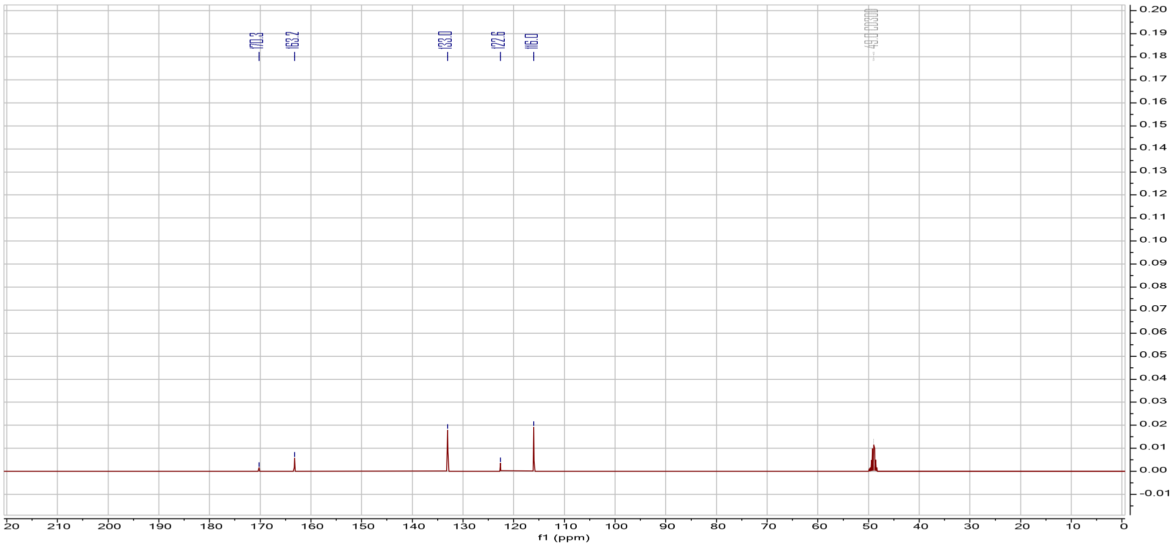

Figure S7. ^{13}C-NMR (100 MHz, CD_3OD) spectrum of compound **3**.

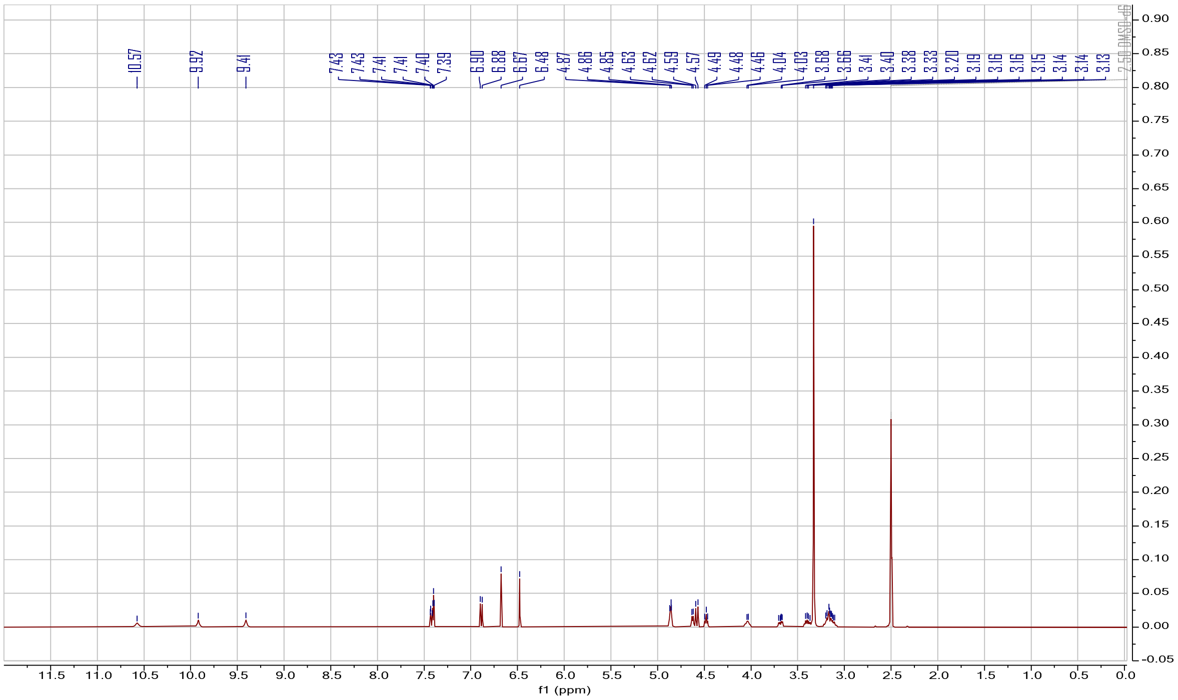

Figure S8. ^{1}H-NMR (400 MHz, CD_3OD) spectrum of compound **4**.

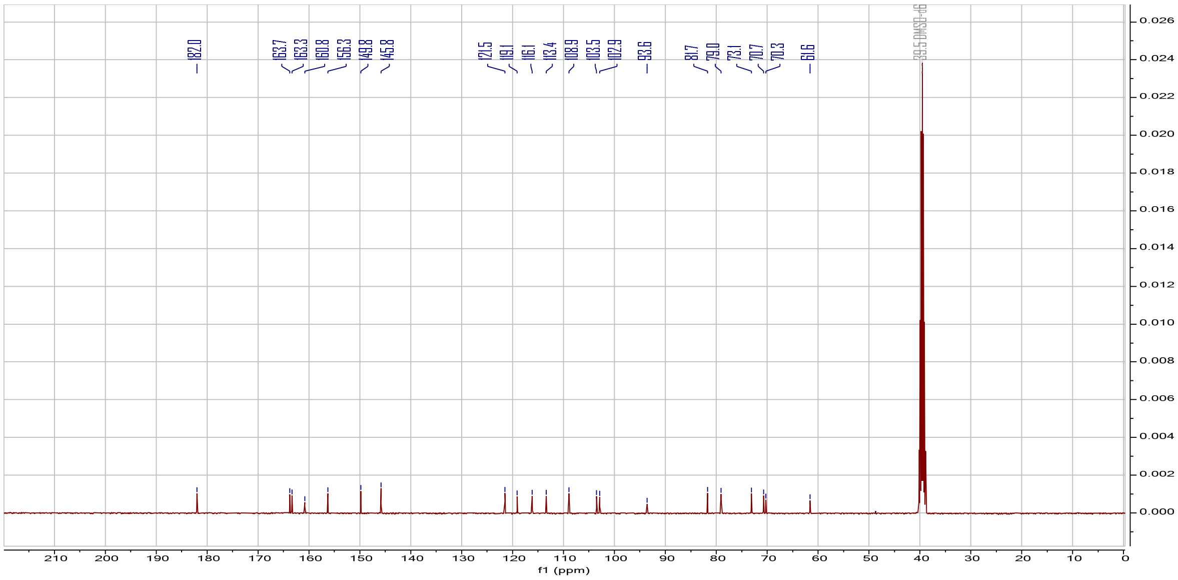

Figure S9. ^{13}C-NMR (100 MHz, CD_3OD) spectrum of compound **4**.

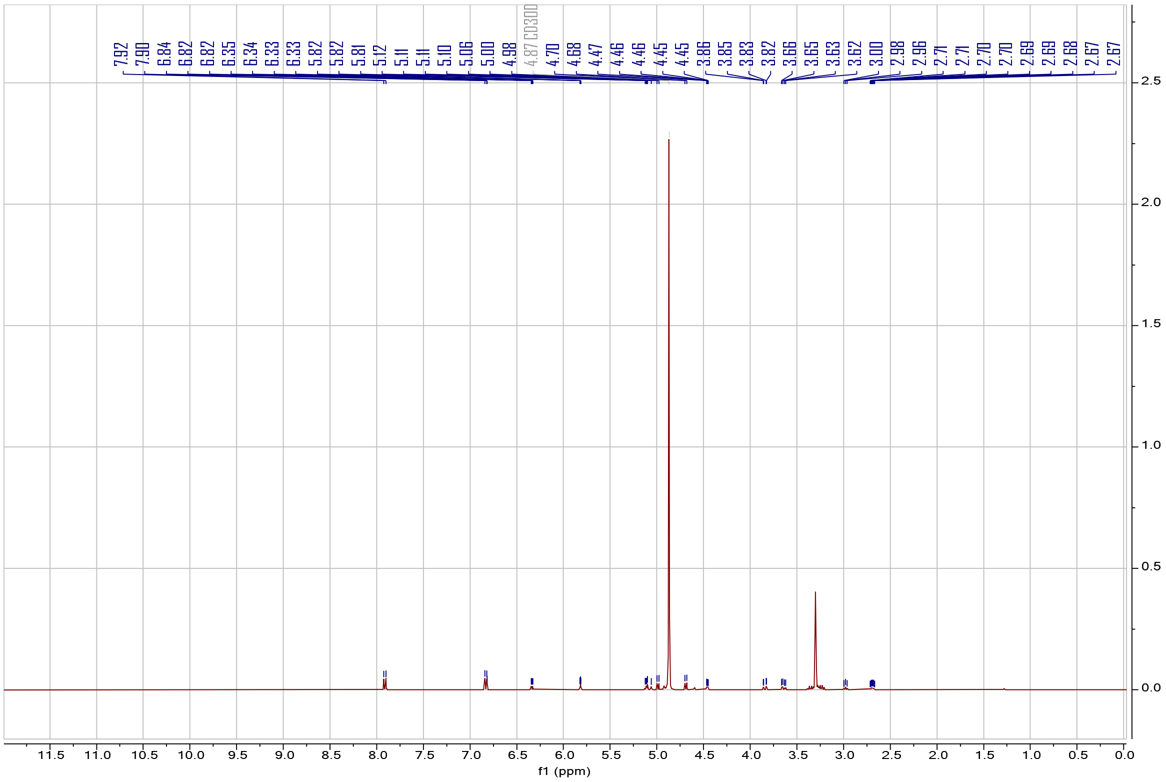

Figure S10. ^{1}H-NMR (400 MHz, CD_3OD) spectrum of compound **5**.

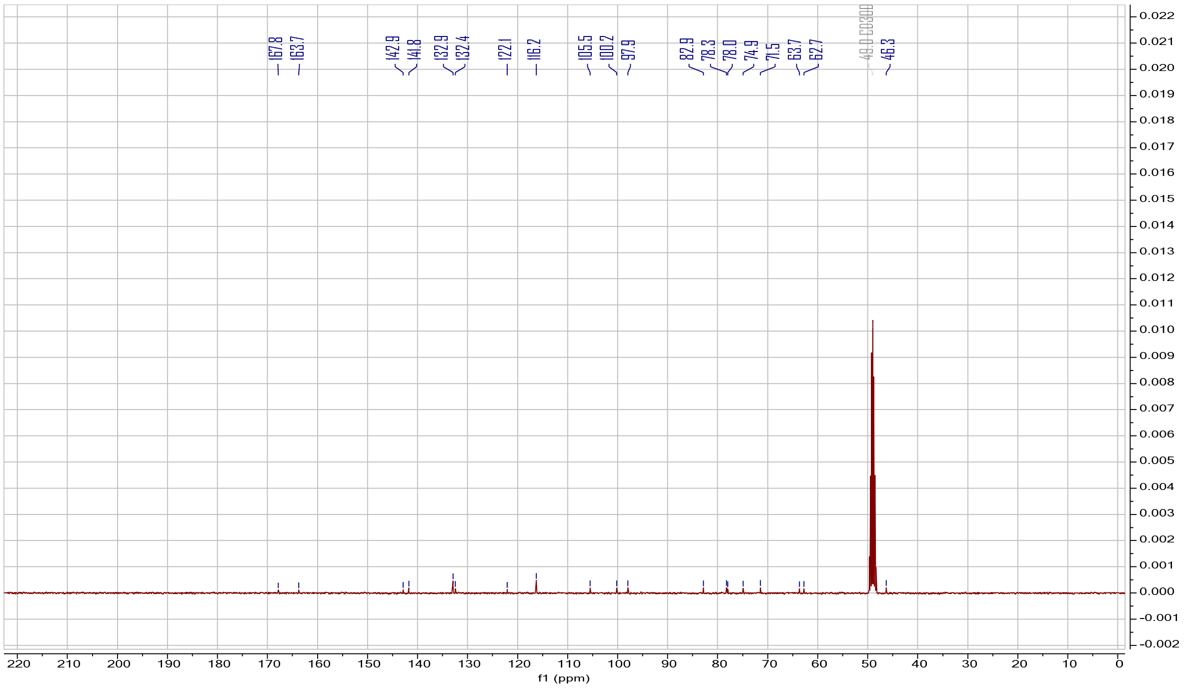

Figure S11. ^{13}C-NMR (100 MHz, CD_3OD) spectrum of compound **5**.

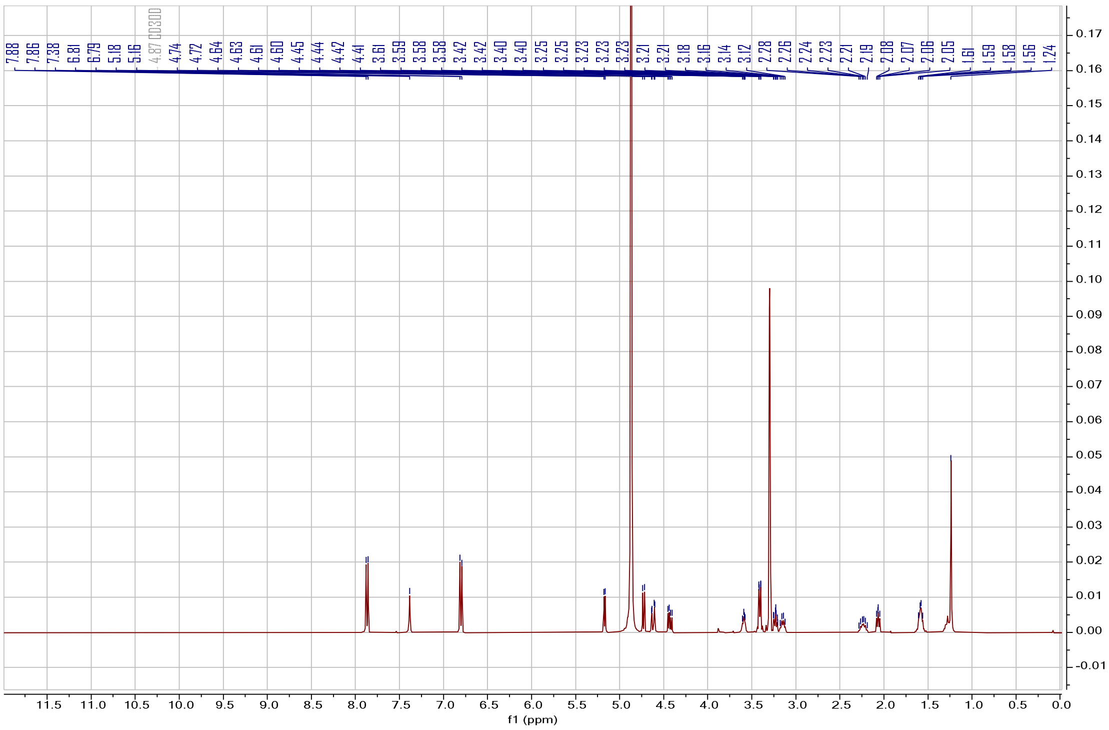

Figure S12. ^{1}H-NMR (400 MHz, CD_3OD) spectrum of compound **6**.

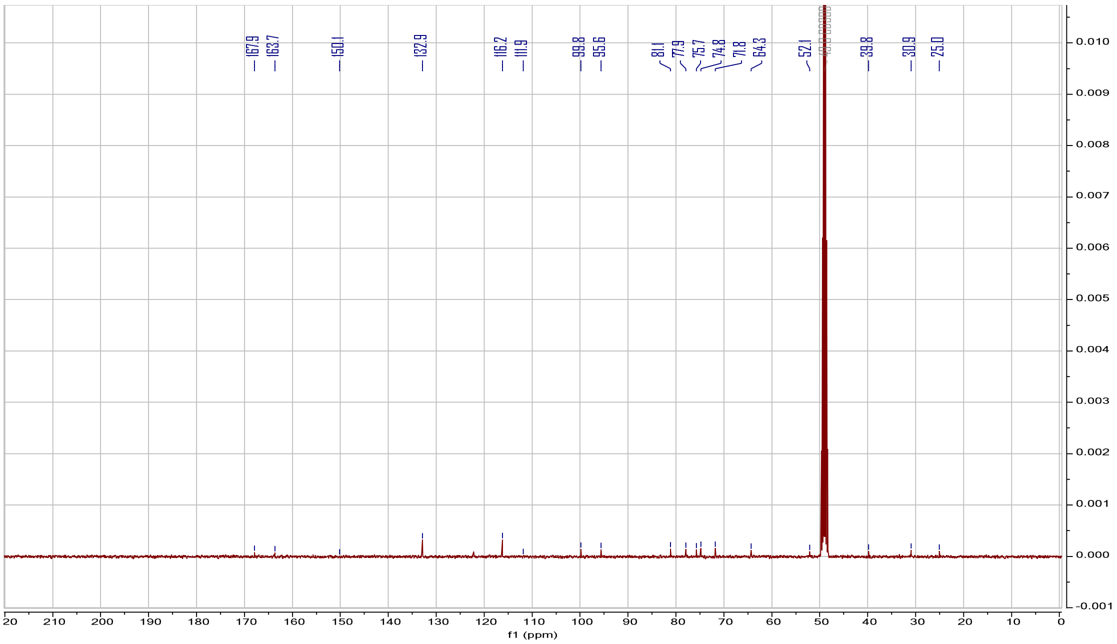

Figure S13. ^{13}C-NMR (100 MHz, CD_3OD) spectrum of compound **6**.

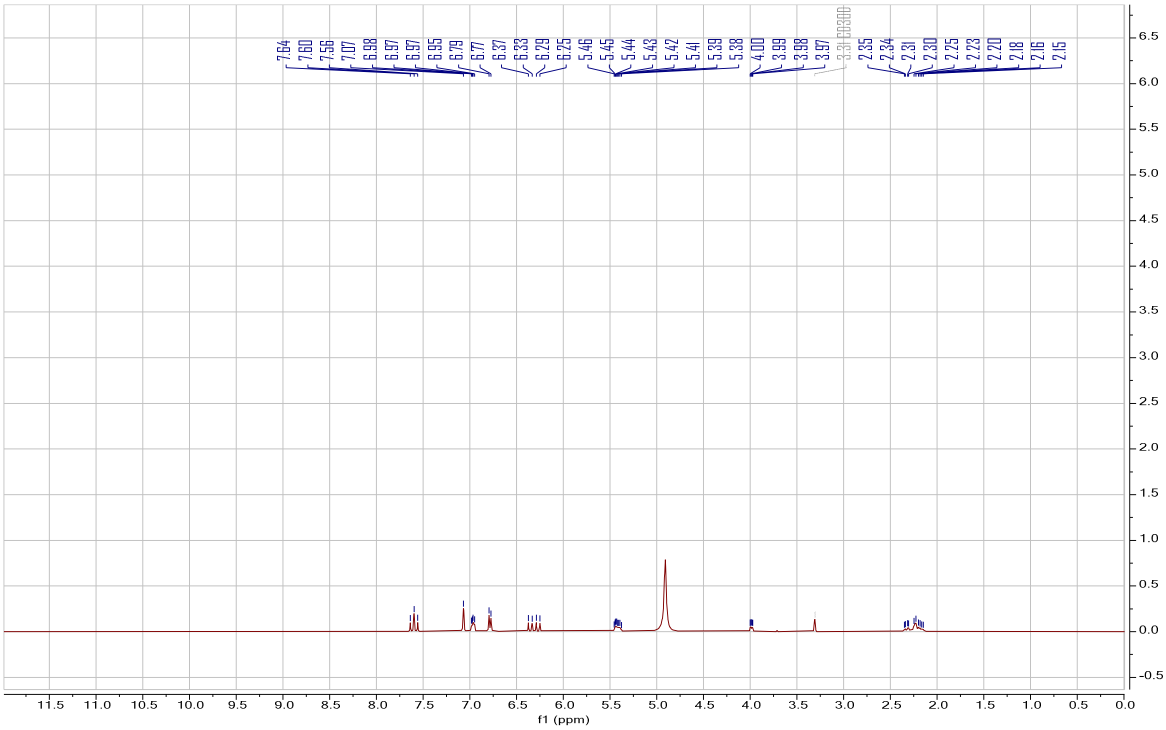

Figure S14. ^{1}H-NMR (400 MHz, CD_3OD) spectrum of compound **7**.

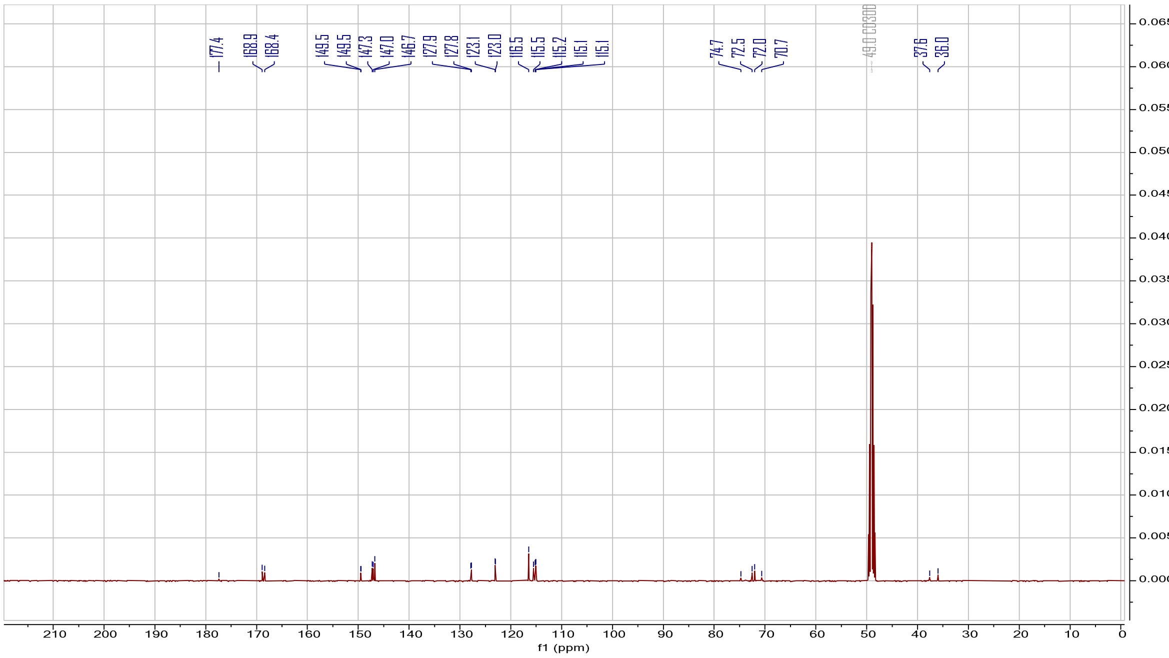

Figure S15. ^{13}C-NMR (100 MHz, CD_3OD) spectrum of compound **7**.

3. Validation method:

Table S1. Retention time and purity of marker compounds (**1**–**7**).

**Compound**	Calibration curve	**Regression Equation**	**RT (min)**	**Purity (%)**	**Correlation Coefficient (r^2)**	**LOD (μg/mL)**	**LOQ (μg/mL)**
Protocatechuic acid (**1**)	0.98 ÷ 125	y = 32515x + 48307	15.1	98.46	0.9995	0.24	0.74
Chlorogenic acid (**2**)	0.98 ÷ 125	y = 10106x + 17177	18.4	97.03	0.9992	0.32	1.18
4-*p*-Hydroxybenzoic acid (**3**)	0.98 ÷ 125	y = 102986x + 168374	19.4	98.01	0.9982	0.35	1.29
Orientin (**4**)	12.5 ÷ 1600	y = 20055x + 13929	22.2	98.04	0.9995	0.37	1.1
Agnuside (**5**)	0.98 ÷ 500	y = 20256x + 23359	23.0	97.58	0.9995	0.15	0.46
6′-*p*-Hydroxybenzoyl-mussaenosidic acid (**6**)	0.95 ÷ 62.5	y = 45543x - 7338	24.2	96.65	0.9984	0.22	0.69
3,5-Dicaffeoylquinic acid (**7**)	0.95 ÷ 62.5	y = 25502x - 10971	27.7	97.52	0.9995	0.25	0.77

4. Bioligical activities:

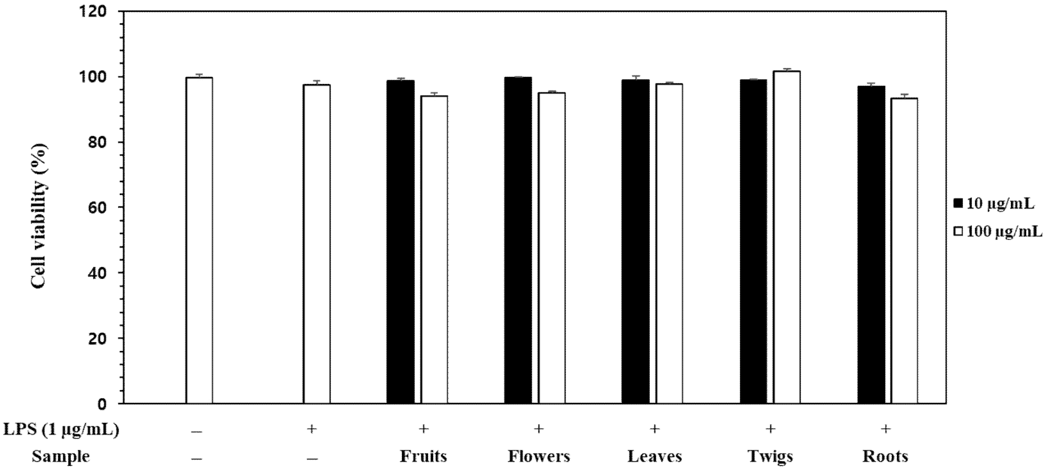

Figure S16. Cell viability of crude extracts on RAW264.7 cells. Negative and positive control were added with/without LPS-stimulation by treatment with medium. The cell viability was expressed as a percentage between the untreated control and treated sample cells. The data are expressed as the mean ± SD (n = 3) of three individual experiments.

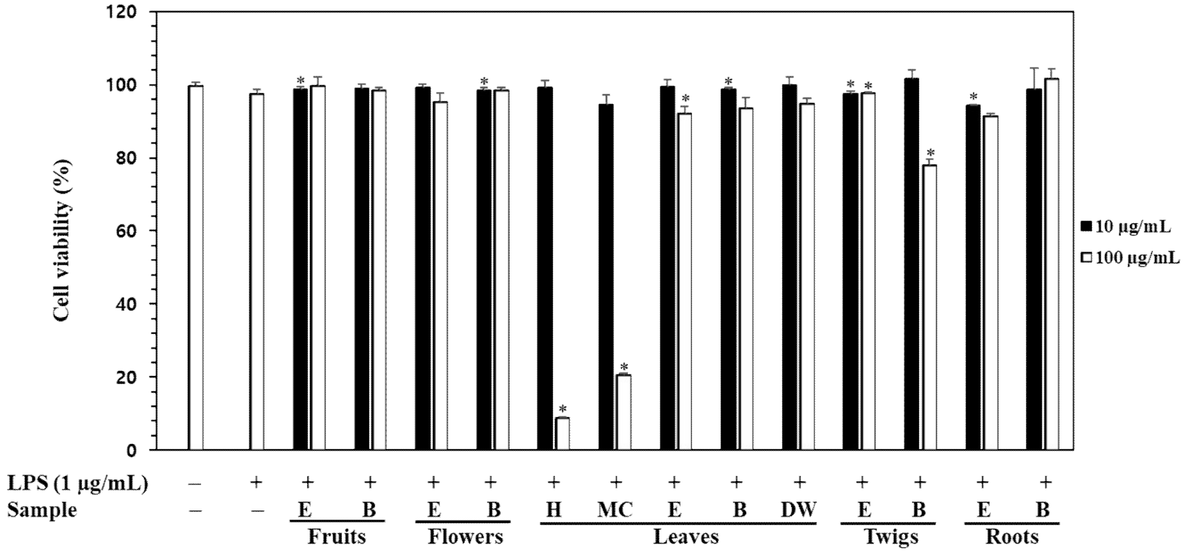

Figure S17. Cell viability of crude extracts and fractions on RAW264.7 cells. Negative and positive control were added with/without LPS-stimulation by treatment with medium. The cell viability was expressed as a percentage between the untreated control and treated sample cells. The data are expressed as the mean ± SD (n = 3) of three individual experiments. $^{*}p < 0.05$.

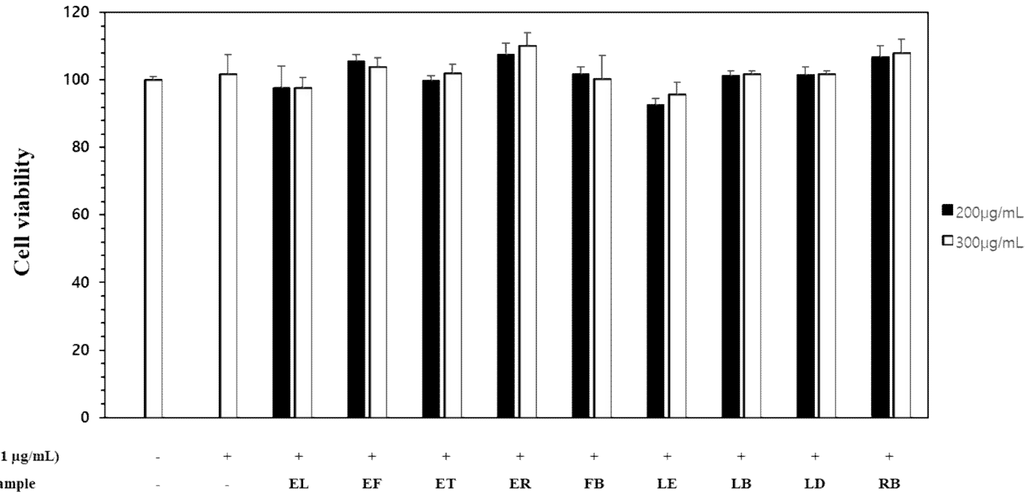

Figure S18. Cell viability of crude extracts and fractions on RAW264.7 cells. Negative and positive control were added with/without LPS-stimulation by treatment with medium. The cell viability was expressed as a percentage between the untreated control and treated sample cells. The data are expressed as the mean ± SD (n = 3) of three individual experiments. $^{*}p < 0.05$.
